# Cell Wall Synthesis, Development of Hyphae and Metabolic Pathways Are Processes Potentially Regulated by MicroRNAs Produced Between the Morphological Stages of *Paracoccidioides brasiliensis*

**DOI:** 10.3389/fmicb.2018.03057

**Published:** 2018-12-11

**Authors:** Juliana S. de Curcio, Juliano D. Paccez, Evandro Novaes, Mathias Brock, Célia Maria de Almeida Soares

**Affiliations:** ^1^Laboratório de Biologia Molecular, Instituto de Ciências Biológicas, Universidade Federal de Goiás, Goiânia, Brazil; ^2^Departamento de Biologia, Universidade Federal de Lavras, Minas Gerais, Brazil; ^3^Faculty of Medicine & Health Sciences, University of Nottingham, Nottingham, United Kingdom

**Keywords:** *Paracoccidioides*, microRNAs, PCM, cell wall, mycosis

## Abstract

MicroRNAs are molecules involved in post-transcriptional gene regulation. In pathogenic fungi, microRNAs have been described at different morphological stages by regulating targets involved in processes such as morphogenesis and energy production. Members of the *Paracoccidioides* complex are the main etiological agents of a systemic mycosis in Latin America. Fungi of the *Paracoccidioides* complex present a wide range of plasticity to colonize different niches. In response to environmental changes these fungi undergo a morphological switch, remodel their cellular metabolism and modulate structural cell wall components. However, the underlying mechanisms regulating the gene expression is not well understood. By using high performance sequencing and bioinformatics analyses, this work characterizes microRNAs produced by *Paracoccidioides brasiliensis*. Here, we demonstrated that the transcript encoding proteins involved in microRNA biogenesis were differentially expressed in each morphological stage. In addition, 49 microRNAs were identified in cDNA libraries with 44 differentially regulated among the libraries. Sixteen microRNAs were differentially regulated in comparison to the mycelium in the mycelium-to-yeast transition phase. The yeast parasitic phase revealed a complete remodeling of the expression of these small RNAs. Analyses of targets of the induced microRNAs, from the different libraries, revealed that these molecules may potentially regulate in the cell wall, by repressing genes involved in the synthesis and degradation of glucans and chitin. Furthermore, mRNAs involved in cellular metabolism and development were predicted to be regulated by microRNAs. Therefore, this work describes a putative post transcriptional regulation, mediated by microRNAs in *P. brasiliensis* and its influence on the adaptive processes of thermal dimorphic fungus.

## Introduction

Small non-coding RNAs play an essential regulatory role in biological systems, without being translated into proteins. Of these RNAs, microRNAs are small RNAs ranging in size from 21 to 24 nt and their action is to post-transcriptionally regulate the expression of target genes involved in different processes such as cell proliferation, tumorigenesis, and infection (Bartel, 2004). The regulation of gene expression by microRNAs is an evolutionarily conserved mechanism, which may have evolved from parasitic infections, since parasites developed strategies to interfere with host microRNA (Hakimi and Cannella, 2011). In fungi, expression of microRNAs is regulated under a wide range of different conditions, such as changes in temperature (Bai et al., 2015) and growth in different morphologies (Zhou et al., 2012; Lau et al., 2013). Several studies have also demonstrated the importance of those molecules during infection of the human host by pathogenic fungi (Croston et al., 2016, 2018).

Despite a current paucity of studies in microRNAs of fungi, data on the importance of those molecules are increasing. In *Penicillium chrysogenum*, the milR-21 was predicted to bind to at least three mRNA targets that are involved in fungal DNA-repair mechanisms. The targets of other microRNAs produced by this fungus include a cytochrome P450 protein, the putative proline-rich call wall protein, PLC-like phosphodiesterase and many hypothetical proteins (Dahlmann and Kück, 2015). MicroRNAs produced by *Aspergillus fumigatus* have target proteins involved in the control of the metabolism, transport, and signal transduction. In addition a mRNA encoding a protein responsible for ustiloxin B biosynthesis is specifically targeted, which binds to tubulin and interferes with the cellular function of microtubules (Bai et al., 2015). MicroRNA produced by *Metarhizium anisopliae*, target mRNAs encoding proteins involved in sporulation and may influence the process of conidia formation (Zhou et al., 2012).

The thermal dimorphic fungus *Penicillium marneffei*, produces microRNAs that target mRNAs encoding proteins such as benzoate 4-monooxygenase, cytochrome P450 and RanBP10, a GTPase involved in the fungus mitosis process (Lau et al., 2013). In addition, microRNAs-like have also been described in vesicles secreted by pathogenic fungi such as *Paracoccidioides brasiliensis, Cryptococcus neoformans, Candida albicans*, and in the non-pathogenic fungus *Saccharomyces cerevisiae*. The presence of these small RNAs in vesicles may be associated with cell signaling processes or even more so in the pathogenesis of these microorganisms (Peres da Silva et al., 2015). Species of the *Paracoccidioides* complex (Matute et al., 2006; Carrero et al., 2008; Teixeira et al., 2009; Turissini et al., 2017), are thermodimorphic fungi that cause systemic mycosis with a high prevalence in Latin America. Countries such as Brazil, Ecuador, and Venezuela have a high incidence of the disease. The disease mainly occurs in rural areas, causing health problems with high mortality rates and often leaving patients with severe sequels (Coutinho et al., 2002; Shikanai-Yasuda et al., 2006, 2017; Bocca et al., 2013; do Valle et al., 2017). To establish an infection, a transtion of fungal mycelium into yeast cells is essential, since strains that are unable to perform this transition are avirulent (De Moraes Borba and Schäffer, 2002).

Studies have demonstrated that species belonging to the *Paracoccidioides* complex, possess plasticity in adapting to different environmental conditions (Nunes et al., 2005; Bastos et al., 2007). Transcriptional studies on individual members of the complex revealed that remodeling of the gene expression during the dimorphic transition event, from mycelium to yeast cells, is a process that precedes the colonization in the host (McEwen et al., 1987). During this event, polysaccharides present in the cell wall are adjusted, including induction of chitin and α-glucan biosynthesis (Bastos et al., 2007). In addition, proteins involved in cellular signaling pathways such as MAPK and calmodulin are induced (Nunes et al., 2005). Other genes have also been described as essential during the dimorphic transition, including histidine kinase *drk1* (Chaves et al., 2016), *Ras-1* and *2* (Fernandes et al., 2008). Blocking the function of the corresponding proteins by specific inhibitors, impairs the dimorphic transition process. In addition, cyclic AMP levels influence the morphogenesis of those fungi, and the PKA inhibitor H99 blocks the dimorphic transition process (Chen et al., 2007; Sestari et al., 2018).

In addition to the morphological changes triggered by temperature in species of the *Paracoccidioides* complex, transcriptional data also demonstrated differential gene expression in each specific stage. Metabolism in the mycelium and yeast cells of *P. lutzii* differs, whereby the mycelium preferably uses an aerobic metabolism, while the yeast cells lean toward alcoholic fermentation, as shown by the increased expression of the alcohol dehydrogenase I encoding gene. In addition, genes of the glyoxylate cycle such as isocitrate lyase are preferentially expressed in the yeast cells (Felipe et al., 2005). In agreement, proteomic data from *P. lutzii* mycelium, the mycelium-to-yeast transition phase and the yeast cells showed an accumulation of enzymes involved in the glycolytic pathway, glyoxylate cycle, and lipid metabolism in the yeast phase. The mycelium proteins related to aerobic energy production and cellular defense against oxidizing agents, such as mitochondrial peroxiredoxin and superoxide, dismutase dependent on manganese were increased. At 22 h of the dimorphic transition of *P. lutzii* from mycelium to yeast, increased levels of heat shock response proteins were detected, as well as proteins of the pentose phosphate pathway. The latter is assumed to produce substrates for glycolysis in the yeast phase that follows subsequently (Rezende et al., 2011). Although, transcriptional and proteomic data reveal a fine-tuned regulation of cellular metabolism and physiology in the specific morphological stages and the dimorphic transition phase, the mechanisms regulating the expression of those genes and proteins has not yet been fully elucidated.

In members of the *Paracoccidioides* species complex, proteins known to be involved in the biogenesis of microRNAs and the silencing of target genes in other fungi, seem to be conserved at least in *P. brasiliensis* (de Curcio et al., in press). Considering the relevance of microRNAs in regulating the essential mechanisms of adaptation and survival, a deeper understanding and knowledge about this species complex is important. Therefore, the objective of this study was to characterize the presence and differential expression of microRNAs in *P. brasiliensis* at different stages of development and to investigate the biological processes regulated by those molecules. By investigating the differential expression of microRNAs, from cDNA libraries, of the different morphological stages, we revealed the presumed role of these molecules, in the adaptation processes of members of the *Paracoccidioides* complex.

## Materials and Methods

### Fungus Strain and Developmental Phases

*P. brasiliensis* (ATCC 32069) in the yeast and mycelium phases was cultivated and maintained in a solid Sabouraud medium (Das et al., 2010) at 36 and 22°C, respectively. The components of the Sabouraud dextrose agar medium (HIMEDIA, M063–500 g) were as follows: Mycological peptone 10% (w/v), dextrose 4% (w/v), 1.5% (w/v) agar. The yeast cells from a 5-day culture on a solid medium were used for the inoculation of the liquid Sabouraud medium and were incubated on a rotary shaker at 150 rpm for 18 h and 36°C. Similarly, the mycelium was collected after 15 days, from a solid medium and transferred to the liquid Sabouraud medium. The mycelium was cultivated under shaking conditions for 18 h at 22°C. For the transition experiments, the mycelium of *P. brasiliensis*, grown in a solid Sabouraud medium for 15 days, was inoculated into the same liquid medium for 96 h at 22°C. A transition of the mycelium into the yeast cells was induced by elevating the incubation temperature of the liquid culture from 22 to 36°C. The transition time of 22 h for subsequent experiments was deduced from counting the number of yeast cells at 0, 22, 48, 72, and 96 h using a Neubauer chamber (Bastos et al., 2007).

### RNAs Integrity, Small RNA Libraries Construction, and Next-Generation Sequencing (NGS)

The mycelial and yeast forms, from 18 h cultures in the liquid Sabouraud medium and cells from the dimorphic transition phase (22 h), were centrifuged at 12,000 × g, for 10 min at 4°C and the supernatant was discarded. Cells were lysed with glass beads in the presence of TRIzol (TRI Reagent, Sigma-Aldrich, St. Louis, MO) and the RNA extraction was performed according to the manufacturer's specifications. The integrity and quality of the RNA samples was evaluated by 1% (w/v) agarose gel electrophoresis, Nanodrop was used (Life Technologies) to check for RNA purity (OD260/ OD280), Qubit ® 2.0 (Life Technologies) for quantification and the 2100 BioAnalyzer (Agilent) system was used to determine the RNA integrity. The RNAs were subsequently stored in a chemically inert matrix GenTegra™ RNA (GenOne Biotechnologies) and sent for sequencing. The mycelium, mycelium-to-yeast transition and the yeast cell libraries were constructed with the NEBNext® Multiplex Small RNA Library Prep Set for Illumina (Illumina KIT). Initially, adapters were ligated to the 3′ and 5′ regions of the RNA molecules, then cDNAs were synthesized, amplified, purified, and the size selected from an agarose gel. The sequencing of the samples was performed by GenOne Biotechnologies Rio de Janeiro-RJ, using the Illumina HiSeq 2500 platform.

### Sequence Analyses for Prediction of MicroRNAs and Secondary Structures

Sequences in FASTQ format were obtained for each library: the mycelium, the mycelium-to-yeast transition cells, and the yeast cells. Initially, the quality of the sequences was evaluated with the FastQC program. Poor quality sequences, as well as the adapters used for library preparation, were removed using the Trimmomatic program (Bolger et al., 2014). After processing the sequences, they were submitted to BLASTx (States and Gish, 1994) against the nr database (https://blast.ncbi.nlm.nih.gov/), to confirm that the sequences of the RNAseq were indeed from *P. brasiliensis*. The processed sequences were then mapped to the reference genome of *P. brasiliensis* present in the NCBI database (https://www.ncbi.nlm.nih.gov/genome/334?genome_assembly_id=212342) using the mapper.pl script of the mirDeep2 program (Friedländer et al., 2012). The output mapping file was analyzed by the miRDeep2.pl script to search for pre-miRNA structures in the *P. brasiliensis* genome. Essentially, mirDeep2 searches for genome locations with many mapped reads and with self-complementary and hairpin structure characteristics of the pre-microRNA (Friedländer et al., 2012). When a pre-microRNA is predicted, mirDeep2 outputs its sequence, including the predicted mature and star sequences in FASTA format. The FASTA file obtained through the mirDeep database, was analyzed against the RNAfold database (http://rna.tbi.univie.ac.at/cgi-bin/RNAWebSuite/RNAfold.cgi) (Lorenz et al., 2011, 2016) in order to identify characteristics of pre-microRNAs such as hairpin, stem-loop and low values of minimum free energy (MFE). The raw sequences were deposited in the NCBI Short Read Archive (SRA) and are available for download under the accession number SRP152968.

### miRNA Differential Expression Analysis by RNA-Seq and Prediction of Target Genes

The numbers of reads mapped to each miRNA were evaluated with the quantifier.pl script of mirDeep2. The result was a counting matrix with each miRNA in the rows and libraries (the mycelium, mycelium-to-yeast transition, and the yeast phases). This count matrix was used for statistical tests of differential expressions among libraries, using DESeq2 (Love et al., 2014), a package of the R/Bioconductor. A likelihood ratio test (LRT) was performed to compare the full model (including all the phases) against the reduced model (only intercept). This LRT was used to find any difference among the phases. In addition, Negative Binomial Wald Tests were performed to compare all three morphological phases in a pairwise manner. The potential targets of miRNA candidates were predicted using 3′ UTR sequences from all *P. brasiliensis* genes. The 3′ UTR sequences were obtained with a custom Perl script that retrieved the first 200 bp sequences from all the transcripts downloaded from the NCBI database (https://www.ncbi.nlm.nih.gov/genome/334?genome_assembly_id=212342) (Brown et al., 2015). The search for homology between the miRNAs and the 3′ UTR of all genes, was performed by the RNAhybrid program (Rehmsmeier et al., 2004), with the following parameters: -f 2,7 for requiring complete complementary alignment at the seed region (positions 2–7 of the microRNA); -e −20 for requiring a minimum free energy of the predicted hybridization below −20 kcal/mol; -*p* 0.05 for a 5% *p*-value cutoff (Lewis et al., 2005). The functional classification of the targets was performed through the Blast2GO (Conesa et al., 2005) and Funcat2 programs.

### Analysis of the Expression of Transcripts by Quantitative Real-Time PCR (qRT-PCR)

Oligonucleotides for the genes involved in biogenesis of microRNAs, such as dicers and argonauts (de Curcio et al., in press) and for targets of microRNAs such as hydrophobin 1 (GenBank XP_010763219), 42 kDa endochitinase (GenBank XP_010763132), cell wall glucanase (Scw4; GenBank XP_010760434), and chitinase 3 (GenBank XP_010761712), were used in the analysis of transcripts (Supplementary Table 1). Total RNA was incubated with DNAse (RQ1 RNase-free DNase, Promega) and subsequently subjected to *in vitro* reverse transcription (SuperScript III First-Strand Synthesis SuperMix; Invitrogen, Life Technologies). Synthesized cDNAs were used in the qRT-PCR reaction using the Step OnePlus platform (Applied Biosystems) with a mixture of SYBR green PCR master mix (Applied Biosystems, Foster City, CA). Normalization of the values was performed using the gene encoding actin (GenBank XP_010761942) (de Curcio et al., in press). Standard curves were generated by a dilution of 1: 5 of the cDNA and the relative expression levels of the transcripts were calculated using the standard curve method for relative quantification (Bookout et al., 2006). Statistical comparisons were performed using the STUDENT's *t*-test and *p* ≤ 0.05 values were considered statistically significant.

## Results

### Morphological and Transcriptional Analyzes and Processing of Data From Sequencing of Small RNA Libraries

The steps used in this study to predict the microRNAs in *P*. *brasiliensis* are shown in the workflow chart (Supplementary Figure 1). The procedure for extracting RNAs of the different phases of *P. brasiliensis* was carried out with the purpose of identifying microRNAs produced by this pathogen. The time of extraction of the RNA in the transition phase from the mycelium-to-yeast cells was previously determined by Bastos et al. (2007), which showed that molecular events precede morphological alterations.

At first, the level of expression of the transcripts encoding proteins involved in the processing of microRNAs, was evaluated in the mycelium, transition from the mycelium-to-yeast and the yeast cells. The transcript coding for Dcr-1 was up-regulated during the transition and in the yeast phase, while Dcr-2 was downregulated during the mycelium-to-yeast transition. The transcript coding for the argonaut Ago-1 was down-regulated during the transition phase, but up-regulated in the yeast phase, whereas the transcript coding for Ago-2 was down-regulated both during the transition and in the yeast phase, although in the yeast phase, the mRNA levels were higher than during the transition phase (Supplementary Figures 2A–D). The differential expression of transcripts encoding proteins related to pre-miRNA processing, suggests that the production of those RNAs could be regulated in the phases of the mycelium, yeast and during the *P. brasiliensis* dimorphic transition. Similar differential regulation of dicers and argonauts was observed in the mycelium and yeast phase of *Penicillium marneffei*. The gene coding for the dicer 2 protein was induced in the mycelium phase and silencing of this gene blocked the synthesis of specific microRNAs of this morphological stage. This demonstrated that induction of these genes correlates to the synthesis and production of mature microRNAs (Lau et al., 2013).

An analysis of microRNAs in different morphological stages of *P. brasiliensis* was performed. The cDNAs were sequenced with the Hiseq2500 Illumina platform in biological triplicates. The number of raw small-RNA sequences obtained ranges from 18,205,091 to 25,256,164 among the different libraries (Table 1).

**Table 1:** Summary of the obtained reads.

**Files^a^**	**Raw reads ^b^**	**Processed reads^c^**	**% Processed reads^d^**	**Small RNA reads mapped to the *Pb* genome^e^**
Mycelium-1	22,324,904	22,319,687	0.02%	1,782,745
Mycelium-2	22,443,544	22,434,973	0.04%	1,778,363
Mycelium-3	21,041,055	21,012,106	0.14%	1,596,263
Transition-1	22,243,452	22,194,102	0.22%	1,614,386
Transition-2	25,225,653	25,146,729	0.31%	1,342,717
Transition-3	25,256,164	25,198,165	0.23%	1,521,910
Yeast-1	18,205,091	18,191,337	0.08%	1,465,015
Yeast-2	19,110,427	19,092,755	0.09%	1,203,138
Yeast-3	19,580,516	19,566,999	0.07%	1,492,691

a Sequence files obtained after RNAseq;

b Total number of sequences obtained;

c Number of sequences remaining after removal of adapters;

d % of sequences removed with the Trimmomatic program;

e *Number of reads mapped in the genome of P. brasiliensis (Pb) with mirDeep2 database*.

An analysis of the quality of reads obtained during sequencing, through the FastQC database, demonstrated sequences of high quality with the vast majority of the bases having PHRED Scores above 30, which indicated an average of one sequencing error per 1,000 base pairs. Supplementary Figure 3 exemplifies the quality of the data of a mycelial cDNA library generated by FastQC. Although the RNAseq data already presented a high quality, additional processing was performed with the Trimmomatic, which was mainly used to remove the attached adapter sequences present in the libraries. The number of sequences for the different conditions, before and after processing, is shown in Table 1. After obtaining the RNAseq data and processing the sequences, they were mapped in the genome of *P*. *brasiliensis*, using the mirDeep2 program.

### Identification of *P. brasiliensis* MicroRNAs by Deep Sequencing and Differential Expression Analyses

**Figure 1:**
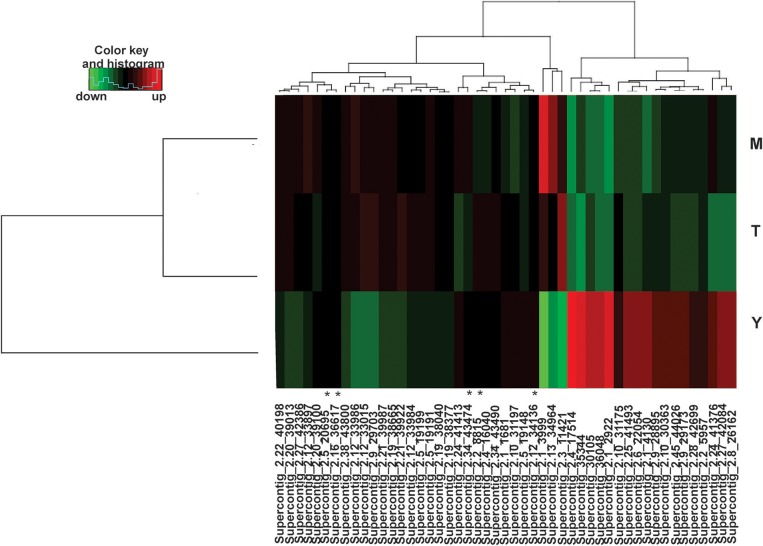
Heat map of identified microRNAs. Among the three libraries 49 microRNAs were identified and 44 were differentially expressed. (^*^) indicates microRNAs without differences in expression in any of the three morphological stages. MicroRNAs differentially expressed are those with values of padj < 0.05. For each library, biological triplicates were generated. Down-regulated microRNAs are shown in green and up-regulated microRNAs in red. (M)-Mycelium, (T)-Transition mycelium-to-yeast cells (Y)-Yeast cells. The DESeq2 a package of the R/Bioconductor was used to group and compare data of expression ratios.

The mapped sequences were also analyzed by miRDeep2 to evaluate the presence of sequences with the potential to be precursors of microRNAs, which would be processed to generate mature microRNAs. Table 2 shows the precursor, mature, and star sequences of the microRNAs among the three analyzed conditions. Among the cDNA libraries, 49 microRNAs were identified with a mirDeep2 score above 5, which was recommended by software to increase the likelihood of true positive predictions. Interestingly, two microRNAs were similar in sequence; those sequences may possibly derive from a tandem duplication in the genome of *P. brasiliensis*. The sequences of all the precursors presented characteristics of hairpin structures with minimum free folding energy values similar to those described in other microorganisms (Jiang et al., 2012; Lau et al., 2013), as depicted in Supplementary Figure 4. Analyses with DESeq2/R indicated that the majority of the predicted microRNAs were differentially expressed among the three morphological phases of the fungus. From the 49 predicted microRNAs, 44 were differentially expressed among the three conditions, with a false discovery rate of 5% (FDR < 0.05). The heat map (Figure 1) shows the expression profile of all 49 microRNAs identified in the cDNA libraries. These differences in the regulation of microRNA expression suggest that such molecules may influence each individual morphological stage.

**Table 2:** MicroRNAs identified in all the cDNA libraries.

**Libraries**	**miRDeep2 score+**	**Mature sequence**	**Star sequence**	**Precursor sequence**
M/T/Y/Supercontig_2.45_44026–	5.20	ugcuguggaaaguuugacug	ugcaagcuuuuugacuucugag	ugcuguggaaaguuugacugucagaaaucugugagauguuuuauuugaagauauuaaauuuauuucucugcaagcuu uuugacuucugag
M/T/Y/Supercontig_2.10_30105+	8.60	uuauuuuuggaacuuuuu	auuaguucggagucugg	auuaguucggagucuggguauggcuuucuuucuguaaugguuuuguuauguuauuuuauuuuuggaacuuuuu
M/T/Y/Supercontig_2.10_30363+	9.00	ugaaaagagaugcacuucagaga	uuugacaauuucuugauuuu	uuugacaauuucuugauuuuguuaauuauuacugaguaacuaaaaaaacagaguuuauuugaaaagagaugcacuuc agaga
M/T/Y/Supercontig_2.14_35344+	13.00	gucagugaaaggauauguauagauu	auuuacacacgacuuccugacuc	gucagugaaaggauauguauagauugaaauguauuuacacacgacuuccugacuc
M/T/Y/Supercontig_2.34_43474+	34.00	gauacaaucacaugucugaga	ucagacaguaacugugu	ucagacaguaacuguguuuauaauaaguuggcagugaucuuugauggcaucuuugacaauaaaaaaauauugagaga uacaaucacaugucugaga
M/T/Y/Supercontig_2.9_29173–	53.00	uaagacagacucugaacagu	uuucauagcaucucuguuggc	uaagacagacucugaacaguuuuuucacuaauauuuuaauaugugaucaugucuuucauagcaucucuguuggc
M/T/Y/Supercontig_2.27_42084+	66.00	auguuuugaacucagaugugu	agagacugacagagcuacagagc	auguuuugaacucagaugugugagaaauuaggcauuaagagaugacucuucacagccuaucacucagagacuga cagagcuacagagc
M/T/Y/Supercontig_2.24_41376–	79.00	aggucugucaagauugaugau	ucagcaaucaagcagcaaguu	ucagcaaucaagcagcaaguugagaugucaucuucucugagacuaucuggaggucugucaagauugaugau
M/T/Y/Supercontig_2.6_22054+	88.00	uagauagaugaauugagcuu	uuuaguauucucgucgcagcu	uuuaguauucucgucgcagcuaucuaguagauagaugaauugagcuu
M/T/Y/Supercontig_2.1_2922–	110.00	gugggcaggcguggaugu	uuuuacuguuugcugacuguugu	gugggcaggcguggaugugggaagcgauuuaauuuuacuguuugcugacuguugu
M/T/Y/Supercontig_2.2_8815–	120.00	ugacuggagaagaugagagga	ugcaucugauucuccaucugu	ugcaucugauucuccaucugucaucuguaugcuguuuucagaggggagaagcagccaacauccaaugacuggaga agaugagagga
M/T/Y/Supercontig_2.22_40198+	150.00	uaucaauuuuauacagacuag	uagucauaaaagaaagcagcu	uaucaauuuuauacagacuaguaagaaagacuauagaguuucuaucucuuauauagucauaaaagaaagcagcu
M/T/Y/Supercontig_2.28_42699–	170.00	aguuggguuguugggcccuagu	uagcuagcuucaaccucaacaaca	aguuggguuguugggcccuaguagccugggcgaccacuccaccgcacgugaugaacccuauauagcuagcuagcuu caaccucaacaaca
M/T/Y/Supercontig_2.13_34964–	290.00	uuuuuucuguuuuucuguuuuu	aauaaaaaaauaaaaaa	aauaaaaaaauaaaaaaucuaaaaaugaaccucaauuucauguuuuuuuuuuuuucuguuuuucuguuuuu
M/T/Y/Supercontig_2.25_41493+	340.00	uguggggcgaggcaguaauugu	ucagccaagcucuauaacagua	uguggggcgaggcaguaauuguggugacugaaaugcuuaaucuuauaccaacaucagccaagcucuauaacagua
M/T/Y/Supercontig_2.4_17514–	390.00	ucagaugaugaaaaagaugcugaca	ucagacuggucucugcuga	ucagacuggucucugcugaaucuccacugcggcaaauggaaguuucagaugaugaaaaagaugcugaca
M/T/Y/Supercontig_2.15_36048+	1100.00	ucugggggggguauggu	uuucuucccuccuggacgaucag	ucugggggggguaugguaauggcuuccucuccggaauugagaauccugucuuuuagugcuuucuucccuccuggac gaucag
M/T/YSupercontig_2.3_11421+	1300.00	uagaagcauaugacccucuacuc	uagcuaucaccaagcucuau	uagcuaucaccaagcucuauaccacaugucugucuagagcauccagucaugcugauggaauagaagcauaugacccu cuacuc
M/T/Y/Supercontig_2.4_16040–	1300.00	ugggagagagcuuauaggga	uuuguauaacaguucug	ugggagagagcuuauagggaugugaucaggauugauuaagauguuuuuguauaacaguucug
M/T/Y/Supercontig_2.34_43490+	1700.00	ugagucugaugagaagcuga	agcaacuuaucagaaugau	ugagucugaugagaagcugaucagcaacuuaucagaaugau
M/T/Y/Supercontig_2.1_1681+	1900.00	uaauccgacgucgaguc	acgugaugaggguuaggccua	acgugaugaggguuaggccuaaucaggcgccgagccuaauccgacgucgaguc
M/T/Y/Supercontig_2.20_39100+	2100.00	auucucugugagcucaguga	uucuucuugcaguaguagc	auucucugugagcucagugauuaucuguugagacuucugcuuugcagagcaguuucugacaaugugaucuuucuucuu gcaguaguagc
M/T/Y/Supercontig_2.12_33015+	2200.00	uaugugcagcaugugaaugu	ugggacugcaugagcauga	uaugugcagcaugugaaugucccaguuuucuacucagagaccugggacugcaugagcauga
M/T/Y/Supercontig_2.27_42386+	2400.00	cuuuacaaaguuggacauuc	ugaacugacucaauggggu	ugaacugacucaauggggugaaauacuuuacaaaguuggacauuc
M/T/Y/Supercontig_2.9_28895+	2600.00	uggauuauaaccaccugcaug	aagcuugaugggaugaa	aagcuugaugggaugaaaggcggugcuggggccugccuggauuauaaccaccugcaug
M/T/Y/Supercontig_2.5_20695–	5000.00	ugggcauaacaugauagac	cuggaauuaguuaucaaaa	ugggcauaacaugauagaccuggucugagccacucuggaauuaguuaucaaaa
M/T/Y/Supercontig_2.1_3999–	5300.00	uagacgugugaguugcucugu	uugaguagacucucgcuucgug	uagacgugugaguugcucuguugacgaaaguuuugaagcuauugcaaguugaguagacucucgcuucgug
M/T/Y/Supercontig_2.5_19148+	5600.00	ugaugcugugcucaaugc	uguugaguguugcauuagaugu	uguugaguguugcauuagauguugaguugaacaucauguuuaaugcuaugucugaugcugugcucaaugc
M/T/Y/Supercontig_2.5_19199+	5600.00	uuacugugguugagaucugu	ugcaauucagcuauaguuu	ugcaauucagcuauaguuucuuauaaaucacugauagaaguugucaaauucuagaaaugauugaacaucuuuuacu gugguugagaucugu
M/T/Y/Supercontig_2.2_5957+	5900.00	uaggccuaaucgggcgccgagc	ucggucgcgcgcugagccu	uaggccuaaucgggcgccgagccuaauccgacgucgagucuaauuaggcgccgggccucggucgggcucggucg cgcgcugagccu
M/T/Y/Supercontig_2.19_38665–	6700.00	ugauaguaauuaacauaugggc	ugugugugaugugcuguugag	ugauaguaauuaacauaugggcaaaggcaugugauaauauacugauuuguuaucucugucugcucaucugucu gugugugaugugcuguugag
M/T/Y/Supercontig_2.12_33897–	7200.00	gaaaaguagucucucuucuua	aagaaugcugcagagauu	gaaaaguagucucucuucuuacuuugaacauuaaaagugcuuuugaugcuguacucagagaaagaaugcugca gagauu
M/T/Y/Supercontig_2.19_38040+	7600.00	ugagguugaaugauucuacuga	uguagaucuaaucucau	ugagguugaaugauucuacugaguguagaucuaaucucau
M/T/Y/Supercontig_2.20_39013+	8600.00	uaucagacugcagaagagaua	aucacauugcacucuguaugaaaga	aucacauugcacucuguaugaaagagaaccacccugagugauaucagacugcagaagagaua
M/T/Y/Supercontig_2.10_31175–	9900.00	uaauacccucacucaucaua	uagguggcugugguguuuga	uaauacccucacucaucauauagguggcugugguguuuga
M/T/Y/Supercontig_2.24_41413–	12000.00	cauuauagaucaugcuucugcagu	ugcuaucaaaaugacugagaug	ugcuaucaaaaugacugagaugagugauuacucauuauagaucaugcuucugcagu
M/T/Y/Supercontig_2.38_43800–	14000.00	uaauucaucugaugcuuucuu	ugaggucagugucagacuga	ugaggucagugucagacugaagcucuauauuaaaauuaacauucuucuguaggagagguaauucaucugaugcu uucuu
M/T/Y/Supercontig_2.10_31197–	14000.00	uauaucaggguguguguuggc	aaccccugauugguaggaaca	uauaucaggguguguguuggcagcaagauauugcaaagagccaaccccugauugguaggaaca
M/T/Y/Supercontig_2.5_19191+	14000.00	ugauaguaauuaacauaugggu	ugugugaugugcuguugaa	ugauaguaauuaacauaugggugauggcaugugacaauauauugauuuguugccucugucugcccaucugucu augugugaugugcuguugaa
M/T/Y/Supercontig_2.12_33986–	15000.00	uaacuguggaaagaagcagu	uguuuccucccaggucaguc	uaacuguggaaagaagcagucucaaugaaguucauauucaucaucuguggugcuguuuccucccaggucaguc
M/T/Y/Supercontig_2.9_29703–	15000.00	uaugguagaggauuuuguga	acaggguguuuugcuugaa	uaugguagaggauuuugugacugaggugcugugggaaguuugacugucagagacccacaggguguuuugcuugaa
M/T/Y/Supercontig_2.1_4130–	19000.00	uaaccaugucgaucugcaga	ucgcggaacccggcagguuggu	ucgcggaacccggcagguuggugcauauauauauguucauuggcgggaagccgggcauuuaagaaugcuguaacc augucgaucugcaga
M/T/Y/Supercontig_2.21_39987–	27000.00	uauucuauucaugcuguuua	cacagacagaaucagauu	cacagacagaaucagauugaaugaacuucugagagagcucuuugauaucccagcagggcuuucacaaggcucaucag ucucaucuauucuauucaugcuguuua
M/T/Y/Supercontig_2.16_36617+	28000.00	ugucauggaucucauaguugag	ucagcucaccacuucugcaaug	ucagcucaccacuucugcaaugcucagaugacugcaagcauuuucuugucauggaucucauaguugag
M/T/Y/Supercontig_2.12_34136–	38000.00	uggacuugauauugcaguuugu	ugaguucucaucaaaucccuuu	ugaguucucaucaaaucccuuucaguggacuugauauugcaguuugu
M/T/Y/Supercontig_2.8_26162+	53000.00	uaagacgcgaacuguuugaggu	uauccaacgguucccauuuggcgu	uaagacgcgaacuguuugagguuucguagaauuauccaacgguucccauuuggcgu
M/T/Y/Supercontig_2.21_39922–	63000.00	uuagggucaauguguggucua	uaccagcagagggacauucuga	uaccagcagagggacauucugaaaucuccggccauccacccagagguuuagggucaauguguggucua
M/T/Y/Supercontig_2.12_33984–	76000.00	uagggucuggacacuucagu	uuguucaguuugaucucuc	uuguucaguuugaucucucuuuguuucacaucugucugaugcucaggcuuagggucuggacacuucagu
M/T/Y/Supercontig_2.19_38377–	7600,00	ugagguugaaugauucuacuga	uguagaucuaaucucau	ugagguugaaugauucuacugaguguagaucuaaucucau

*+ Score mirdeep: Log-odds score assigned to the hairpin by mirDeep2*.

### MicroRNAs in *P. brasiliensis* Are Differentially Regulated in the Fungus Phases

Comparing cDNA libraries from the mycelium and the mycelium-to-yeast transition, 16 microRNAs were differentially expressed, with seven induced in the mycelium and nine in the transition phase (Supplementary Table 2). By comparing the microRNAs present in the yeast cells and in the transition phase, 38 microRNAs were differentially expressed, whereby 18 were induced during the mycelium-to-yeast cell transition and 20 were more abundant in the yeast cells (Supplementary Table 3). Comparisons between the mycelium and the yeast cells identified 39 microRNAs differentially regulated, 19 were induced in the yeast cells and 20 in the mycelium (Supplementary Table 4).

### Potential Targets for MicroRNAs in *P. brasiliensis* Morphological Phases

**Figure 2:**
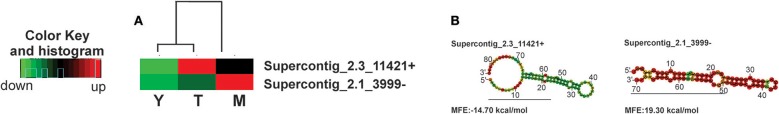
Heat map of microRNAs with the highest values of expression in the mycelium and transition and the predicted secondary structures. **(A)** Heat-map, **(B)** predicted secondary structure. Down-regulated microRNAs are shown in green and up-regulated microRNAs in red. (M)-Mycelium, (T)-Transition mycelium-to-yeast cells (Y) yeast. The secondary structure of the pre-microRNAs was predicted by the RNAFOLD database.

To analyze the processes regulated by microRNAs in the different morphological phases, we investigated the targets of the microRNAs. Due to the large number of microRNAs differentially regulated in the cDNA libraries, we first focused on microRNAs with high expression values in the mycelium and the transition phase, but low expression in the yeast cells. We found two up-regulated microRNAs in Supercontig_2.3_11421 and Supercontig_2.1_3999 with Padj > 0.05 and a log2 fold change >4. MicroRNAs that did not fit these criteria were not used in future analyses. Figure 2A depicts the heat map of the selected microRNAs and the predicted secondary structures are shown in Figure 2B. By analyzing the cellular processes that could be regulated by these microRNAs, we identified GO-terms such as biogenesis of cellular components, cell rescue, defense, virulence and metabolism (Supplementary Figure 5A,B, respectively). Some of the likely proposed targets of these microRNAs are depicted in Table 3. In agreement with different metabolic preferences in the mycelium and yeast cells, proteins involved in β-oxidation such as acyl-CoA synthetases and enoyl-CoA hydratase/isomerase were targets of microRNAs induced in the mycelium and the transition phase. Other gene targets of microRNAs up-regulated in the mycelium and transition libraries include genes involved in the oxidative stress response such as thioredoxin and glutathione-S-transferase. Coinciding with that, several studies already demonstrated that these genes are preferentially expressed in the yeast cells rather than in the other morphological stages of this fungus (Marques et al., 2004; Nunes et al., 2005). Furthermore, a GPI anchored serine-threonine rich protein was identified as a target, which putatively belongs to the PbPga1family of *P. brasiliensis*. This protein is present in the yeast cell surface at the septum between the mother cell and the bud and was therefore expected to be downregulated in the mycelium and during the transition phase while RNA levels from *PbPga1* were higher in yeast cells compared to the mycelium (Valim et al., 2012).

**Table 3:** Biological processes regulated by differentially expressed microRNAs.

**MicroRNA**	**Up-regulated**	**Down-regulated**	**Selected target**
**BIOGENESIS OF CELLULAR COMPONENTS**			
Supercontig_2.1_2922	Yeast	Mycelium/Transition	PADG_07744/42 kDa endochitinase
Supercontig_2.14_35344	Yeast	Mycelium/Transition	PADG_04922/cell wall glucanase (Scw4)
Supercontig_2.25_41493	Yeast	Mycelium/Transition	PADG_05303/beta-1,6-glucan biosynthesis protein (Knh1),
Supercontig_2.15_36048	Yeast	Mycelium/Transition	PADG_02143/cell wall biogenesis Mhp1
Supercontig_2.15_36048	Yeast	Mycelium/Transition	PADG_06374/chitinase 3
Supercontig_2.25_41493	Yeast	Mycelium/Transition	PADG_04274/polysaccharide synthase Cps1p
Supercontig_2.3_11421	Mycelium/Transition	Yeast	PADG_03015/GPI anchored serine-threonine rich protein
**CELL FATE**			
Supercontig_2.1_2922	Yeast	Mycelium/Transition	PADG_07875/hydrophobin 1
**CELLULAR COMMUNICATION/SIGNAL TRANSDUCTION MECHANISM**			
Supercontig_2.15_36048+	Yeast	Mycelium/Transition	PADG_04598/small G- GPA2
**ENERGY**			
Supercontig_2.3_11421	Mycelium/Transition	Yeast	PADG_08572/acyl-CoA synthetases
Supercontig_2.1_3999	Mycelium/Transition	Yeast	PADG_06425/ enoyl-CoA hydratase/isomerase
Supercontig_2.1_3999	Mycelium/Transition	Yeast	PADG_04495/coenzyme A synthetase
**CELL RESCUE, DEFENSE, AND VIRULENCE**			
Supercontig_2.1_3999	Mycelium/Transition	Yeast	PADG_00697/glutathione S-transferase
Supercontig_2.1_3999	Mycelium/Transition	Yeast	PADG_02118/chaperone heat shock hsp12 protein
Supercontig_2.1_3999	Mycelium/Transition	Yeast	PADG_03161/thioredoxin

**Figure 3:**
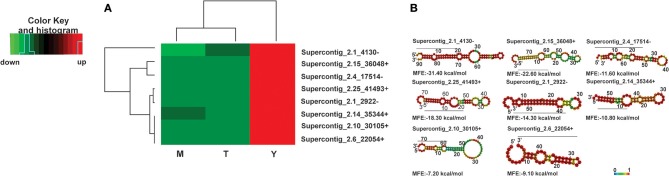
Heat map of microRNAs with the highest expression values in the yeast cells and predicted secondary structures **(A)** Heat-map, **(B)** secondary structure. Down-regulated microRNAs are shown in green and up-regulated microRNAs in red. (M)-Mycelium, (T)-Transition mycelium-to-yeast cells, (Y) yeast. The secondary structure of the pre-microRNAs was predicted by the RNAFOLD database.

We then analyzed the biological processes that might be regulated by the microRNAs that were induced specifically in the yeast cells. MicroRNAs with higher differential expression in the yeast cells possess values of Padj < 0.05 and log^2^ fold changes >4. The expression profile and structure of these selected microRNAs are shown in Figures 3A and 3B while those that are regulated by some of these microRNAs are shown in the Supplementary Figures 5C–J. The predicted targets of the bioinformatics analysis, are involved in the synthesis and degradation of polymers present in the cell wall, for example chitinase 3, cell wall glucanase (Scw4p), and 42 kDa endochitinases. In addition, microRNAs that target the mRNAs of proteins involved in the development of hyphae, cell division, and morphogenesis, were identified. Examples inlcude Gpa2p and hydrophobin (Table 3).

### Target Confirmation and Overview of Processes Regulated by MicroRNAs

**Figure 4:**
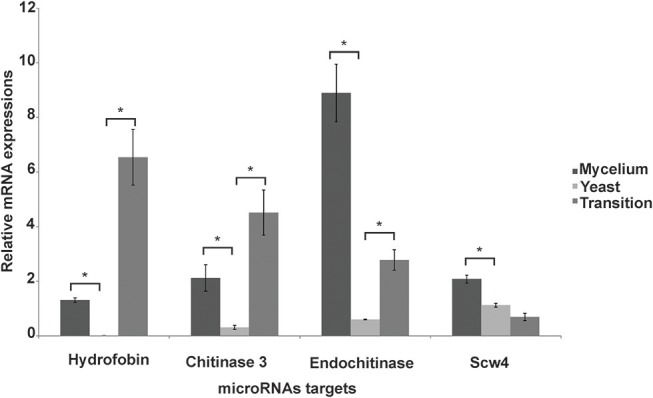
Analysis of expression of target genes of microRNAs induced in the yeast cells. Quantitative RT-PCR was performed on the mycelium, transition from mycelium to yeast and the yeast cells. Data were normalized against the actin gene (GenBank XP_010761942). Data are expressed as mean ± standard deviation from triplicates. A statistically significant difference was determined by Student's *t*-test, (^*^) represents *p* ≤ 0.05.

Some of the targets, that can possibly be silenced by microRNAs induced in the yeast phase, included the cell-wall related chitinase 3, cell wall glucanase (Scw4p), 42 kDa endochitinase, and the mycelium-associated hydrophobin as depicted in Table 3. To confirm that the target mRNAs were indeed down-regulated in the respective morphological phase, qRT-PCR was performed (Figure 4). Indeed, the potential targets for yeast cell induced microRNAs decreased in the yeast cells. This reinforces the assumption that microRNAs may be specifically involved in the down-regulation of the target genes in the parasitic phase of this pathogen.

## Discussion

Proteins involved in the processing of microRNAs are conserved in different kingdoms. Here, we found that the expression of the dicer and argonaut encoding genes *dcr*-1, *dcr*-2, *ago*-1, and *ago*-2 was differentially regulated among the different morphological phases of *P. brasiliensis*. *dcr-2* and *ago-2* was more expressed in the mycelium phase, whereas *dcr-1* dominates in the yeast cells. These results agree with an investigation on *P. marneffei*, a human thermodimorphic pathogenic fungus. In this fungus, expression of the mRNAs coding for *dcr*2 and *ago*-2 revealed a significant increase in the mycelium compared to the yeast cells. In contrast, *dcr*-1 mRNA expression was induced in the yeast cells rather than the mycelium. This is consistent with previous studies on *Neurospora crassa* and *P*. *marneffei* (Lee et al., 2010; Lau et al., 2013) that showed different dicer proteins were involved in the processing of microRNAs. For example, the miRNAs PM-milR-M1 and PM-milR-M2 from *P*. *marneffei* are only produced in the filamentous phase and processing is dependent on the dicer 2 protein, while dicer 1 is not essential. Our analyses on the differential expression of dicers and argonaut genes indicate that the same mechanism of microRNA processing might also be true for *P*. *brasiliensis*.

The presence of microRNAs among different morphological stages of *P. brasiliensis* was analyzed by high performance DNA sequencing from cDNA libraries and obtained between 25,256,164 to 18,205,091 raw reads, from which 1,203,138 to 1,782,745 were mapped in the genome of *P. brasiliensis*. These data are consistent with those obtained for other fungi (Zhou et al., 2012; Lau et al., 2013) and confirm the high quality of our data. The subsequent analyses of the mapped reads allowed the identification of 49 microRNAs in the cDNA libraries that showed the expected characteristics of pre-microRNAs such as hairpin, stem loop and low values of free energy folding (Bartel, 2004). From the 49 identified microRNAs, 44 were differentially regulated. This number is significantly higher than that described for *P. marneffei*, in which 17 microRNAs were detected in the mycelium and seven in the yeast phase (Lau et al., 2013). In the mycelium and the conidia of the enthomopathogenic fungus *Metarhizium anisopliae*, 11 microRNAs were identified of which, six microRNAs were common to both conditions. It has been speculated that the differential expression of microRNAs between the two morphological stages could influence the development of this fungus, as many targets of those microRNAs are proteins related to sporulation (Zhou et al., 2012). This assumption is supported by the microRNA targets from *P. brasiliensis* identified in this study.

One essential process in the establishment of Paracoccidioidomycosis in the host, is the ability of *P. brasiliensis* to survive the elevation of the temperature and to respond to oxidative agents. Targets of microRNAs induced the mycelium and the mycelium-to-yeast transition phase are glutathione S-transferase, thioredoxin, and chaperone heat shock hsp12 protein, which indicates that their production is specifically suppressed in conditions other than the yeast cells. In agreement, Nunes et al. (2005) showed that the corresponding genes are preferentially up-regulated in the yeast cells and the proteins are important for survival within the macrophages and to counteract free oxygen radicals that are more detrimental at 37°C than at the low-temperature saprophytic condition. Similar results were also described by Marques et al. (2004). As these microRNAs were only induced in the mycelium and in the first stages of dimorphic transition, a possible role in regulating the response to increasing temperatures and oxidizing agents, is very likely.

Several microRNAs were also found to be specifically induced in yeast cells. During the infection process and the accompanied morphological switch, species of the *Paracoccidioides* complex changed the composition of polysaccharides in the cell wall, whereby the cell wall of the yeast cells is mainly composed of chitin and α-1,3-glucan (Kanetsuna and Carbonell, 1970). In agreement, the microRNA present in the supercontig_2.1_2922, targets the 42 kDa endochitinase. Studies on *P.lutzii* revealed that the CTS1p endochitinase is induced during the back-transition from the yeast into the mycelium, in order to remove chitin from the cell wall, which favors the formation of hyphae in the filamentous phase (Bonfim et al., 2006). Chitinase 3 was also found as a microRNA target in *P. brasiliensis* yeast cells. Accordingly, in *P*. *lutzii* proteomic studies showed that chitinase 3 was only detected in the cell wall of the mycelium (Araújo et al., 2017). Similarly, in *Candida albicans* the chitinase activity is higher in hypha and production, and hydrolysis of chitin was induced during yeast-hypha morphogenesis (Selvaggini et al., 2004). Therefore, the induction of chitinase-suppressing microRNAs in parasitic yeast cells of *P. brasiliensis* might possibly result in increased chitin concentrations, as its degradation is inhibited. In addition to a microRNA targeting chitinase 3 and endochitinase, we also identified microRNAs targeting a putative cell wall glucanase (Scw4p), which presents a high similarity to the *A. fumigatus* cell wall glucanase Scw4p and the glycosyl hydrolase 17 family from *S. cerevisiae*. In the pathogenic fungus *A. fumigatus*, a mutation in the gene encoding the Scw4p, results in a decrease of the β-glucan in the cell wall as well as a reduction of filamentous growth and increased sensitivity to cell wall stressors, possibly due to the alteration in the constitution of the cell wall polymer (Millet et al., 2018). Transcript and protein expression levels of the β-1,3-glucanosyltransferase of the glycosyl hydrolase 17 family (GEL3p) in *P. lutzii*, specifically increased in the mycelium phase (Castro Nda et al., 2009). β-1,6-glucan biosynthesis (Knh1) and cell wall biogenesis (Mhp1) proteins may also be silenced by microRNAs induced in the yeast phase. In *S*. *cerevisiae*, the Mhp1 protein is involved in the synthesis of β-1-6-glucans (Lai et al., 1997) and this polymer is present in the cell wall of yeast cells only in small amounts (Puccia et al., 2011). In the fission yeast *Schizosaccharomyces pombe*, the Bgs1p/Cps1p was identified as a putative beta 1,3-glucan synthase and phenotypic analyses demonstrated its involvement in β 1,3 glucan synthesis, polarized growth and germination of spores. As β-1,3-glucan is the main polysaccharide of the mycelium phase in *P. brasiliensis*, the induction of proteins involved in the biosynthesis of this polymer is not required for yeast cells and should be down-regulated, due to its high immunogenic potential (Kanetsuna and Carbonell, 1970; Mendes et al., 2017). In summary, microRNAs seem to contribute to the fine-tuning of cell wall remodeling, by a possible induction or repression of the proteins involved in polymer biosynthesis in the different morphological stages.

Production of hydrophobins was also found to be suppressed by microRNAs induced in the parasitic yeast form. Hydrophobins are small hydrophobic proteins, involved in different cellular processes such as cell growth and development in fungi. In *A*. *fumigatus* (RodA) hydrophobin is involved in the permeability, hydrophobicity and immune-inertia of conidia (Valsecchi et al., 2017). Similarly, in *Paracoccidioides* spp. the mRNA from hydrophobins 1 and 2 were detected only in the mycelial phase and during the first 24 h of the mycelium to yeast transition (Goldman et al., 2003; Albuquerque et al., 2004). In agreement with the possible regulatory role, the microRNA targeting hydrophobin 1 was repressed in the mycelial and dimorphic transition.

The formation of pseudo-hyphae in *S. cerevisiae*, during nitrogen deprivation involves the Ras2p, which activates adenylyl cyclase to produce cyclic adenosine monophosphate. Furthermore, a Gpa2p belonging to the G-protein family, is involved in the development of pseudo-hyphae in *S*. *cerevisiae* and mutations in this gene blocked the formation of pseudo-hyphae and reduced levels of intracellular cyclic AMP (Kübler et al., 1997; Lorenz, 1997). In *P. brasiliensis* the microRNAs targeting Gpa2p were induced in the parasitic yeast phase but were repressed in the filamentous phase and during the dimorphic transition. This can be seen as a control mechanism to prevent the development of hyphae.

**Figure 5:**
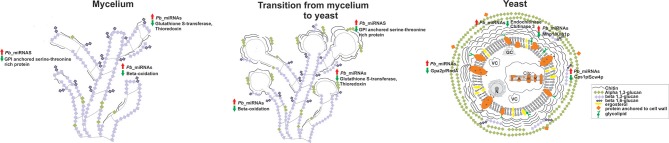
Predicted targets for microRNAs that were induced between the mycelium, transition of mycelium to yeast, and the yeast cells cDNA libraries. Cell wall constitution, morphogenesis, formation of hyphae, response to oxidizing agents, and β-oxidation are some of the processes putatively regulated by microRNAs. Red arrows indicate up-regulated microRNAs and green arrows indicate down-regulated proteins and biological processes by microRNAs. N, cell nucleus; VC, vacuole; GC, Golgi complex.

Figure 5 provides a brief overview on the pathways that are possibly regulated by microRNAs, in the three morphological phases, predicted by bioinformatics tools. MicroRNAs induced in the mycelium and the dimorphic transition libraries, are assumed to regulate genes involved with β-oxidation and those involved in the response to oxidative agents, temperature increase, and cell wall morphology changes. On the contrary, microRNAs induced in the yeast phase which have putative target proteins involved in the degradation, could silence the chitin and synthesis of β-1,3 glucan and β-1,6 glucan. These yeast cell induced microRNAs additionally silence the synthesis of proteins involved in hyphae formation, such as Gpa2p. Therefore, *P. brasiliensis* perhaps employs the production of microRNAs as a mechanism of post-transcriptional gene regulation, that favors the morphological, metabolic, and adaptive changes carried out by this fungus, in order to promote infection.

## Author Contributions

CS and JdC conceived and designed the experiments. JdC performed the experiments. JdC, EN, MB, and CS analyzed and/or interpreted the data. CS contributed to reagents and materials. JdC, JP, EN, MB, and CS analyzed the data and wrote the manuscript.

### Conflict of Interest Statement

The authors declare that the research was conducted in the absence of any commercial or financial relationships that could be construed as a potential conflict of interest.

## Supplementary Material

Supplementary Figure 1 Work flow chart Stages for characterization of microRNAs in the mycelium, mycelium-to-yeast transition, and yeast cells from cDNA libraries.

Supplementary Figure 2 Analysis of expression of genes encoding proteins involved in post-transcriptional gene silencing. qRT-PCR on **(A)** *dcr-1*, (GenBank XP_010761164); **(B)** *dcr-2* (GenBank XP_010762450); **(C)** *ago-1*, (GenBank XP_010755946); **(D)** *ago-2* (GenBank XP_010758266) in the mycelium, mycelium-to-yeast, and the yeast cells. The data were normalized against the transcript encoding actin (GenBank XP_010761942). The *T*-test was used for statistical comparisons. Error bars represent standard deviation of three biological replicates. (^*^) Represents *p* ≤ 0.05.

Supplementary Figure 3 Distribution of Phred quality score of sequences from the library M1_raw.fg. The majority of the sequences, for every read position presented Phred score > 30. Phred quality scores indicate the probability, in log scale (Phred = –log p), that a given base has been correctly identified during sequencing. A Phred score of 30 indicates 1 error for every 1,000 bases sequenced. For each position, within all reads, there is a yellow boxplot indicating the distribution of Phred score for that read position.

Supplementary Figure 4 Secondary structure of the identified microRNAs. The secondary structure of the microRNAs and the negative free energy values were predicted using the RNA fold tool. The hairpin structures were common to all pre-microRNAs. Predicted values of MFE for pre-microRNAs of *P. brasiliensis* were similar to those already described for other pre-microRNAs present in fungi. The structures are colored according to the base pairing probabilities. Red color denotes high probability, as represented in the color bar.

Supplementary Figure 5 Biological processes regulated by microRNAs in *P. brasiliensis*. Biological processes regulated by differentially expressed microRNAs that presented a fold change with log^2^ > 4.0.

Supplementary Table 1 Oligonucleotide sequences used in the present study.

Supplementary Table 2 Differentially expressed microRNAs between the mycelial and transition libraries.

Supplementary Table 3 Differentially expressed microRNAs between the transition and yeast libraries.

Supplementary Table 4 Differentially expressed microRNAs between the mycelium and yeast libraries.
