# Supplementary figures and images for: Cell Wall Synthesis, Development of Hyphae and Metabolic Pathways Are Processes Potentially Regulated by MicroRNAs Produced Between the Morphological Stages of Paracoccidioides brasiliensis
Source: Front Microbiol. 2018 Dec 11;9:3057. doi: 10.3389/fmicb.2018.03057 (PMC6297277; doi:10.3389/fmicb.2018.03057)

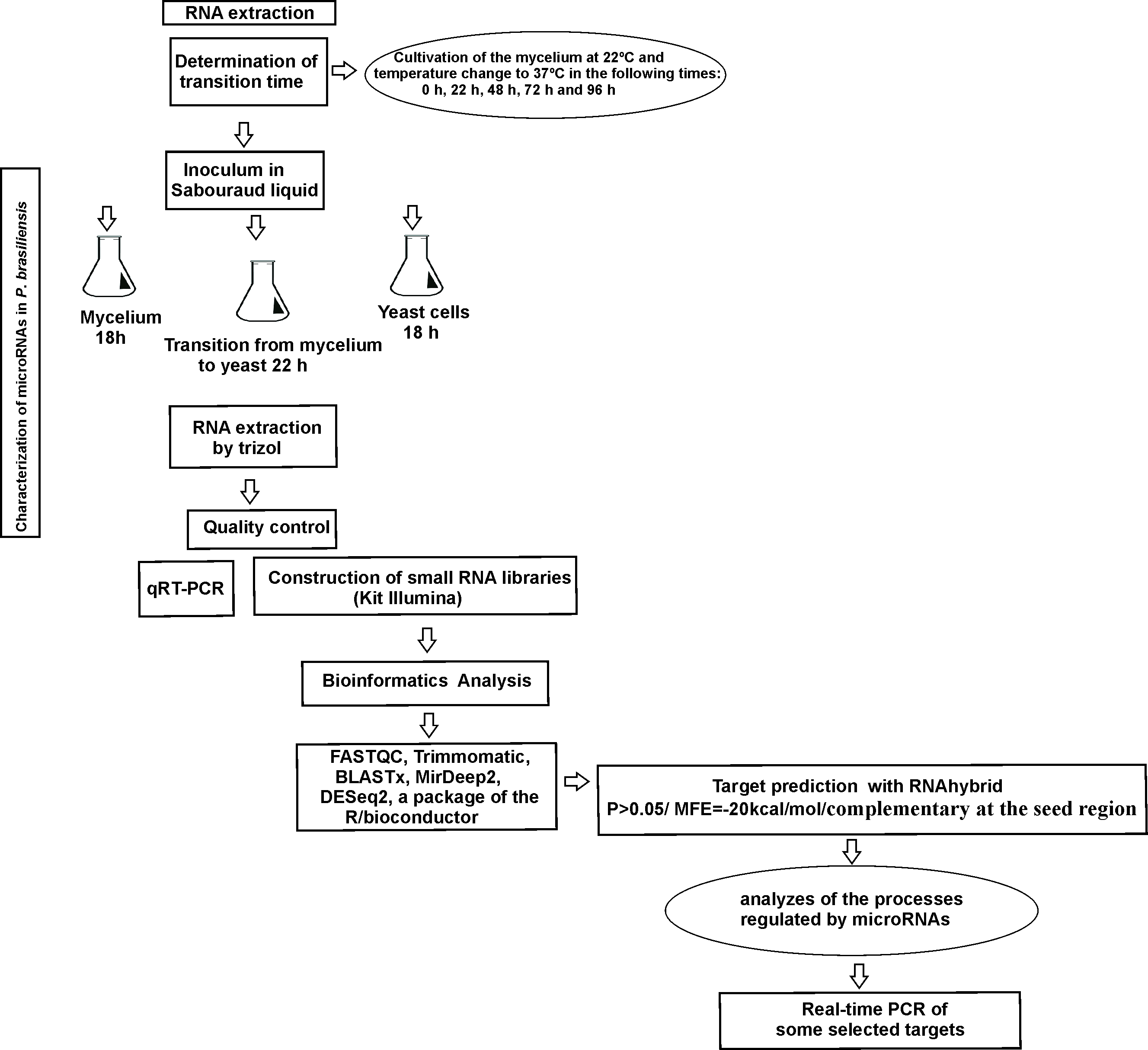

Supplement: Supplementary Figure 1 — Work flow chart Stages for characterization of microRNAs in the mycelium, mycelium-to-yeast transition, and yeast cells from cDNA libraries. [file Image_1.TIF]

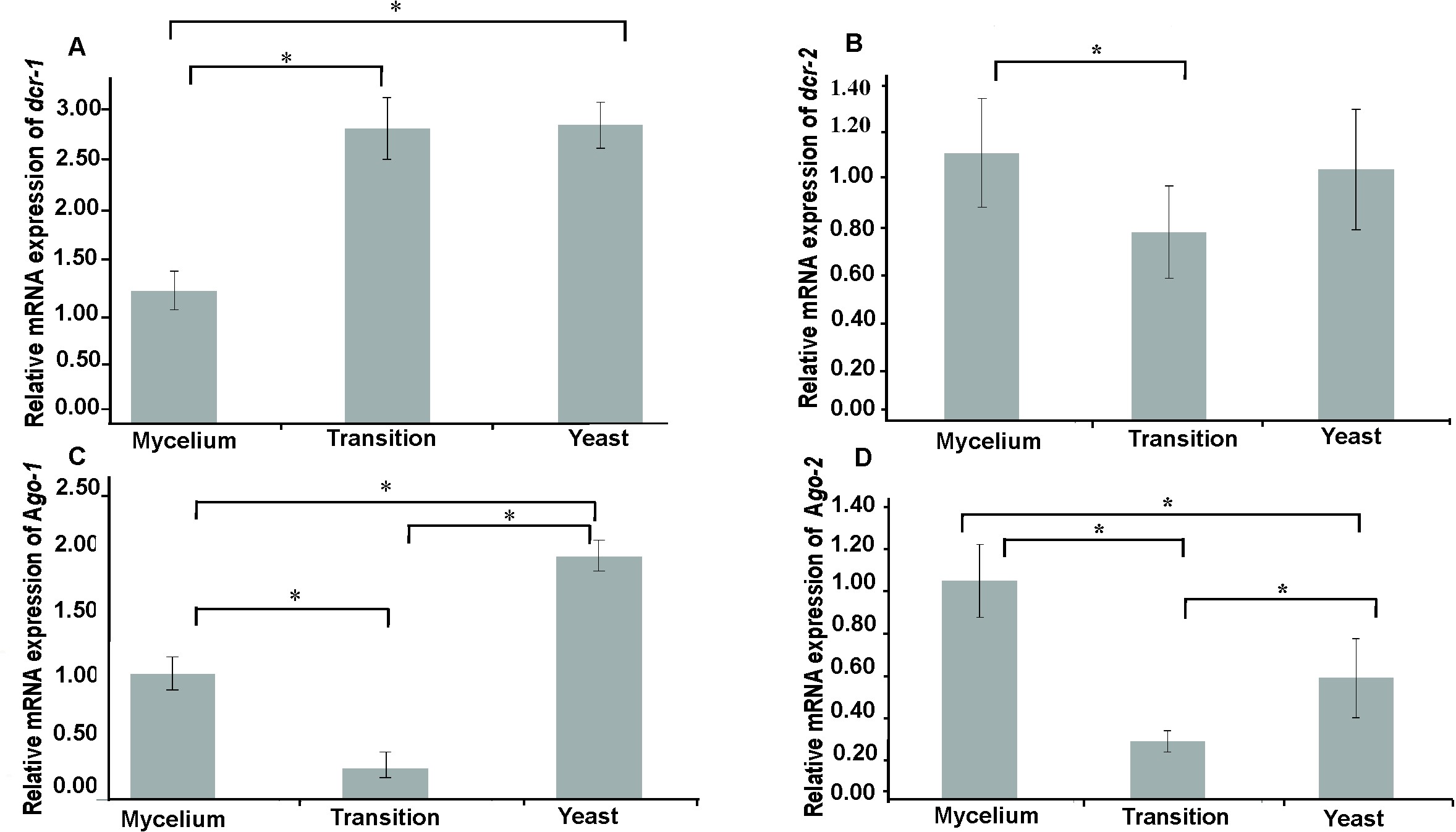

Supplement: Supplementary Figure 2 — Analysis of expression of genes encoding proteins involved in post-transcriptional gene silencing. qRT-PCR on (A) dcr-1, (GenBank XP_010761164); (B) dcr-2 (GenBank XP_010762450); (C) ago-1, (GenBank XP_010755946); (D) ago-2 (GenBank XP_010758266) in the mycelium, mycelium-to-yeast, and the yeast cells. The data were normalized against the transcript encoding actin (GenBank XP_010761942). The T-test was used for statistical comparisons. Error bars represent standard deviation of three biological replicates. (*) Represents p ≤ 0.05. [file Image_2.TIF]

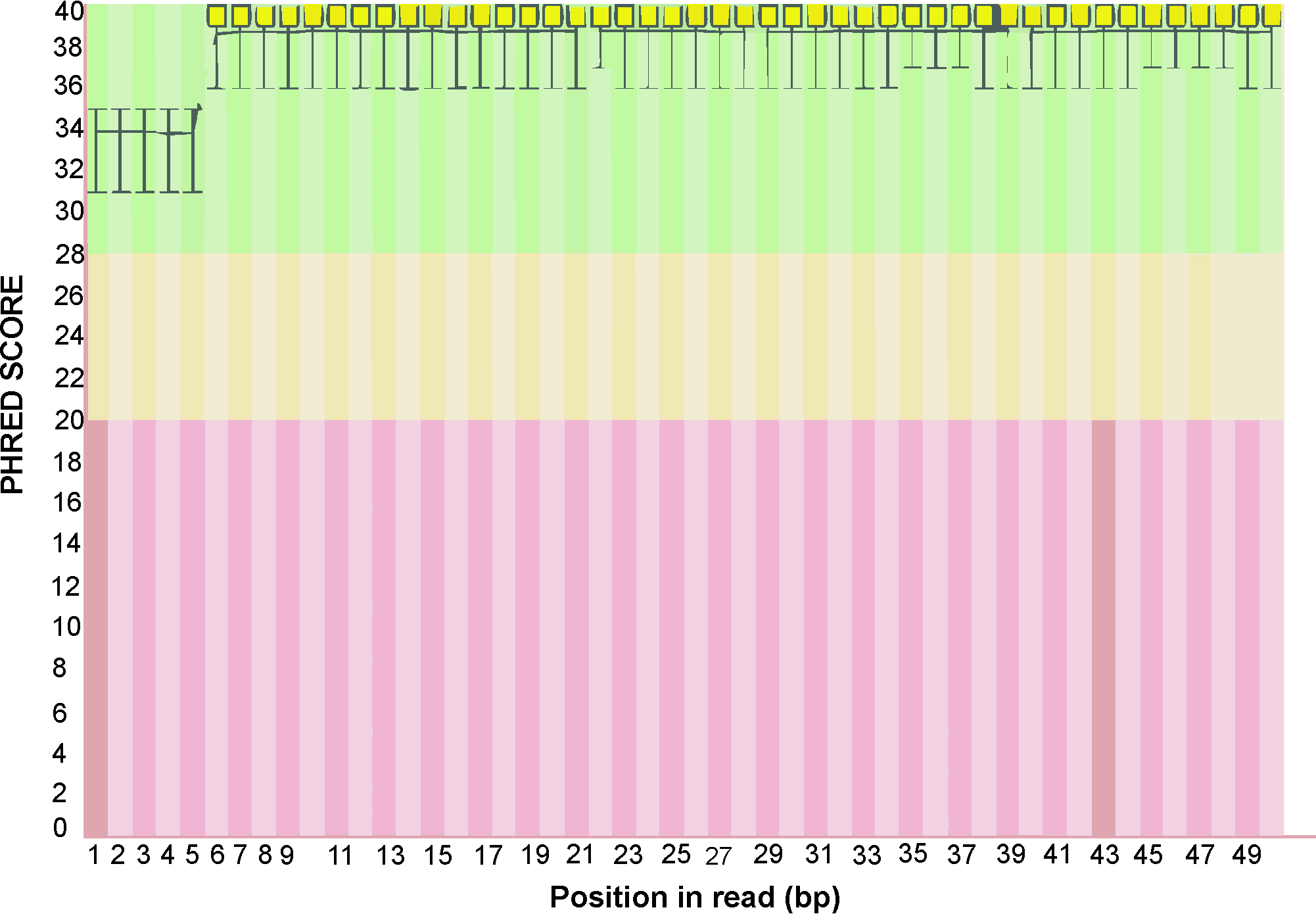

Supplement: Supplementary Figure 3 — Distribution of Phred quality score of sequences from the library M1_raw.fg. The majority of the sequences, for every read position presented Phred score > 30. Phred quality scores indicate the probability, in log scale (Phred = –log p), that a given base has been correctly identified during sequencing. A Phred score of 30 indicates 1 error for every 1,000 bases sequenced. For each position, within all reads, there is a yellow boxplot indicating the distribution of Phred score for that read position. [file Image_3.TIF]

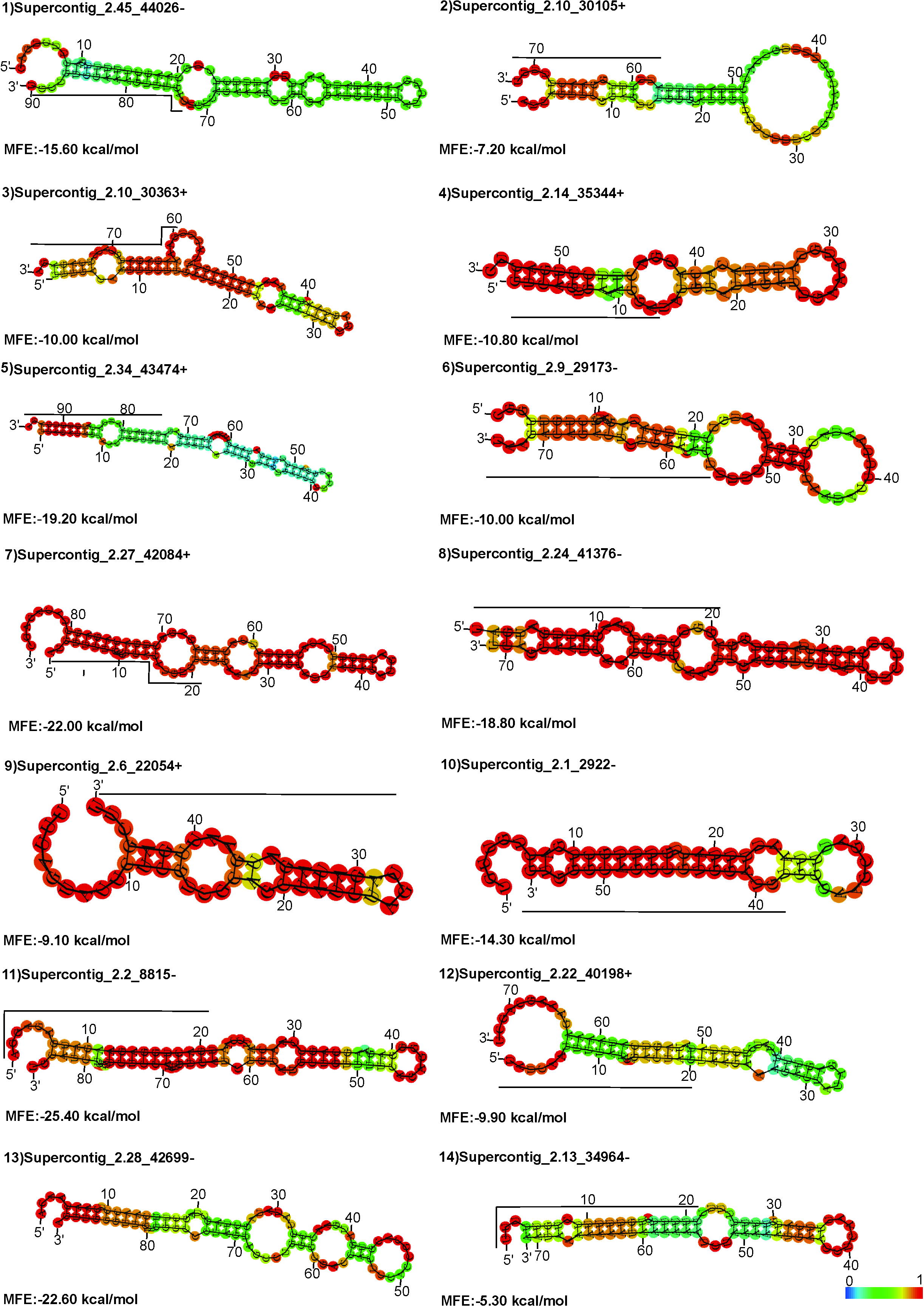

Supplement: Supplementary Figure 4 — Secondary structure of the identified microRNAs. The secondary structure of the microRNAs and the negative free energy values were predicted using the RNA fold tool. The hairpin structures were common to all pre-microRNAs. Predicted values of MFE for pre-microRNAs of P. brasiliensis were similar to those already described for other pre-microRNAs present in fungi. The structures are colored according to the base pairing probabilities. Red color denotes high probability, as represented in the color bar. [file Image_4.TIF]

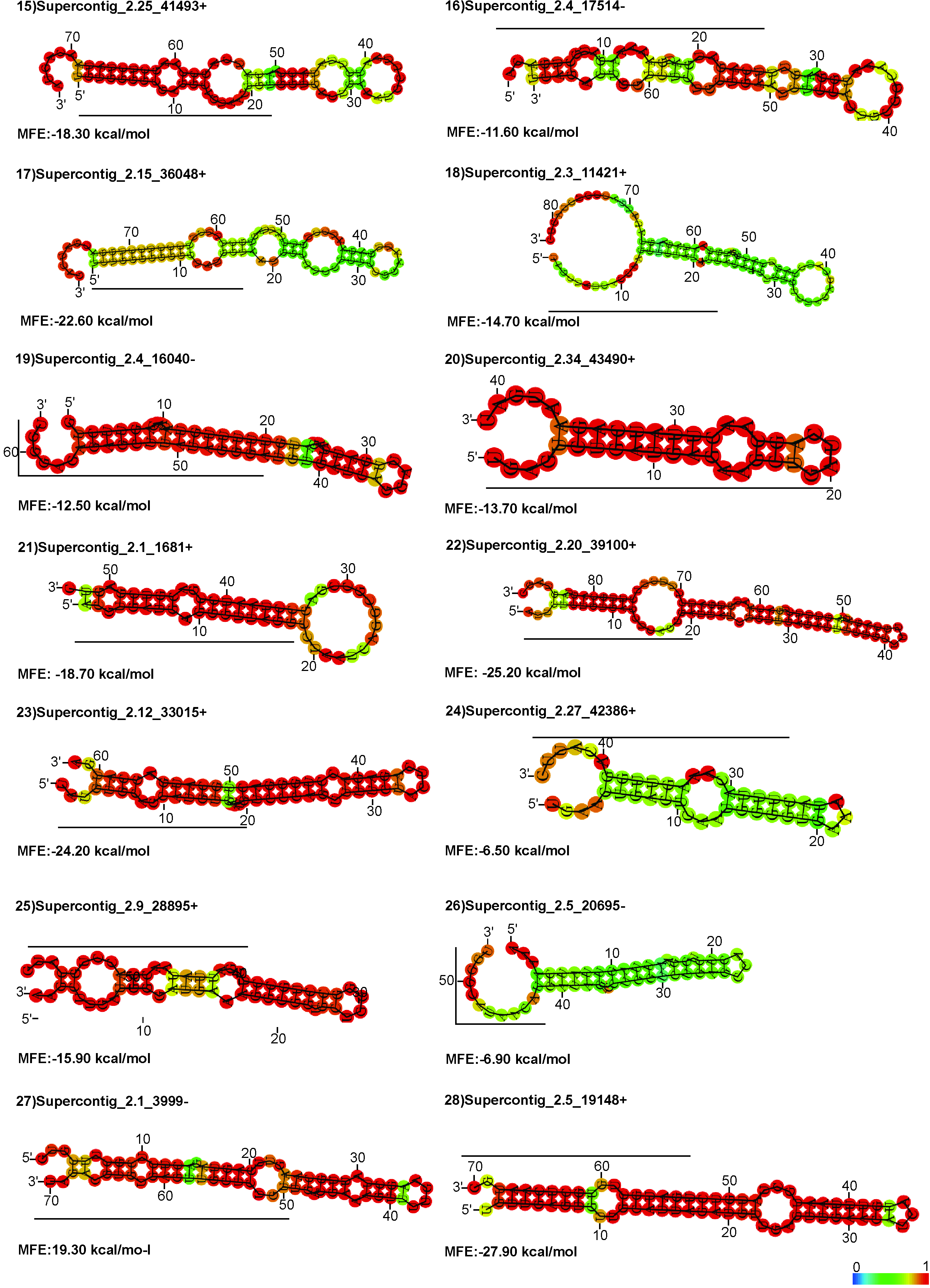

Supplement: Supplementary Figure 5 — Biological processes regulated by microRNAs in P. brasiliensis. Biological processes regulated by differentially expressed microRNAs that presented a fold change with log2 > 4.0. [file Image_5.TIF]

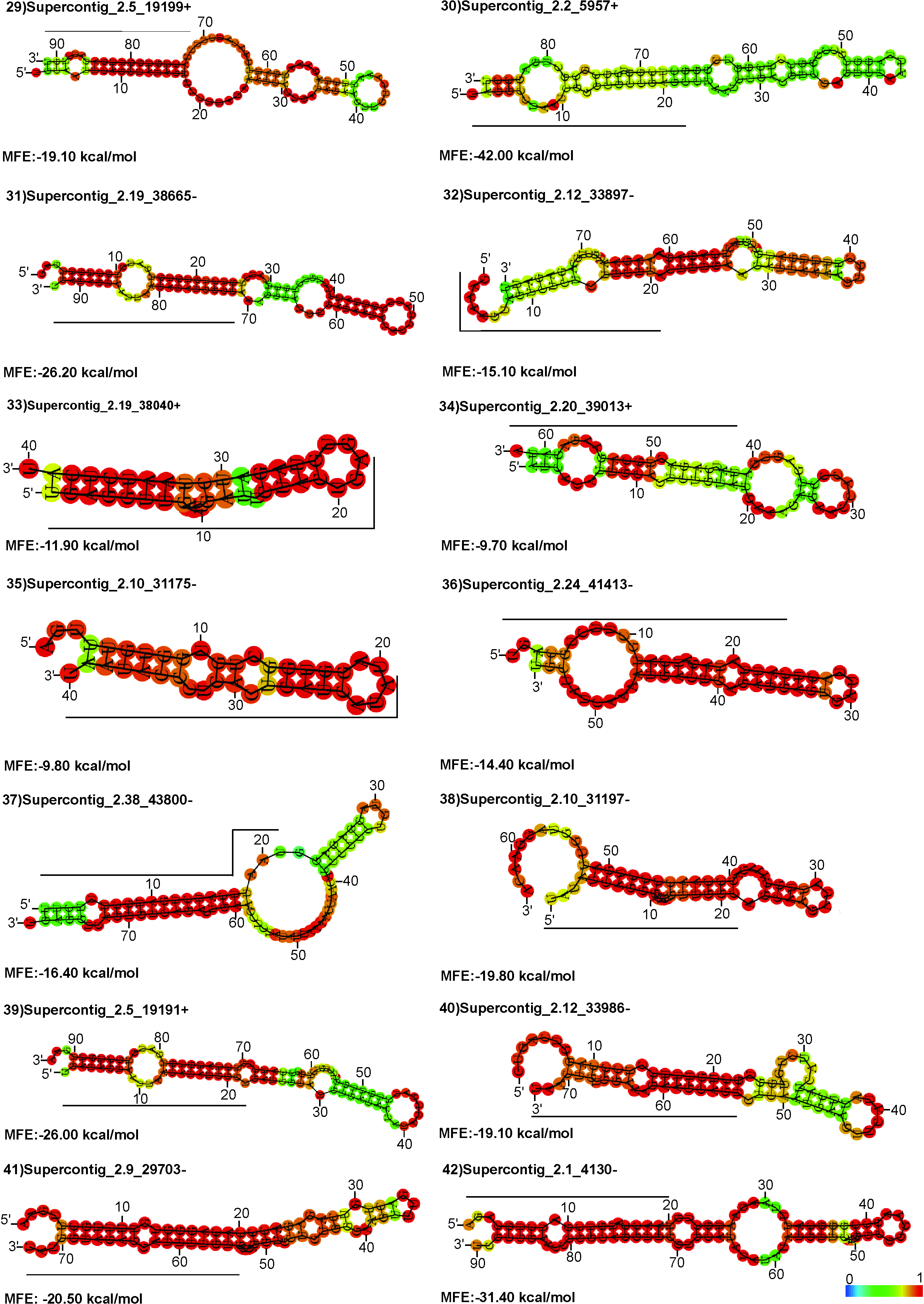

Supplement: Supplementary file 10 [file Image_6.TIF]

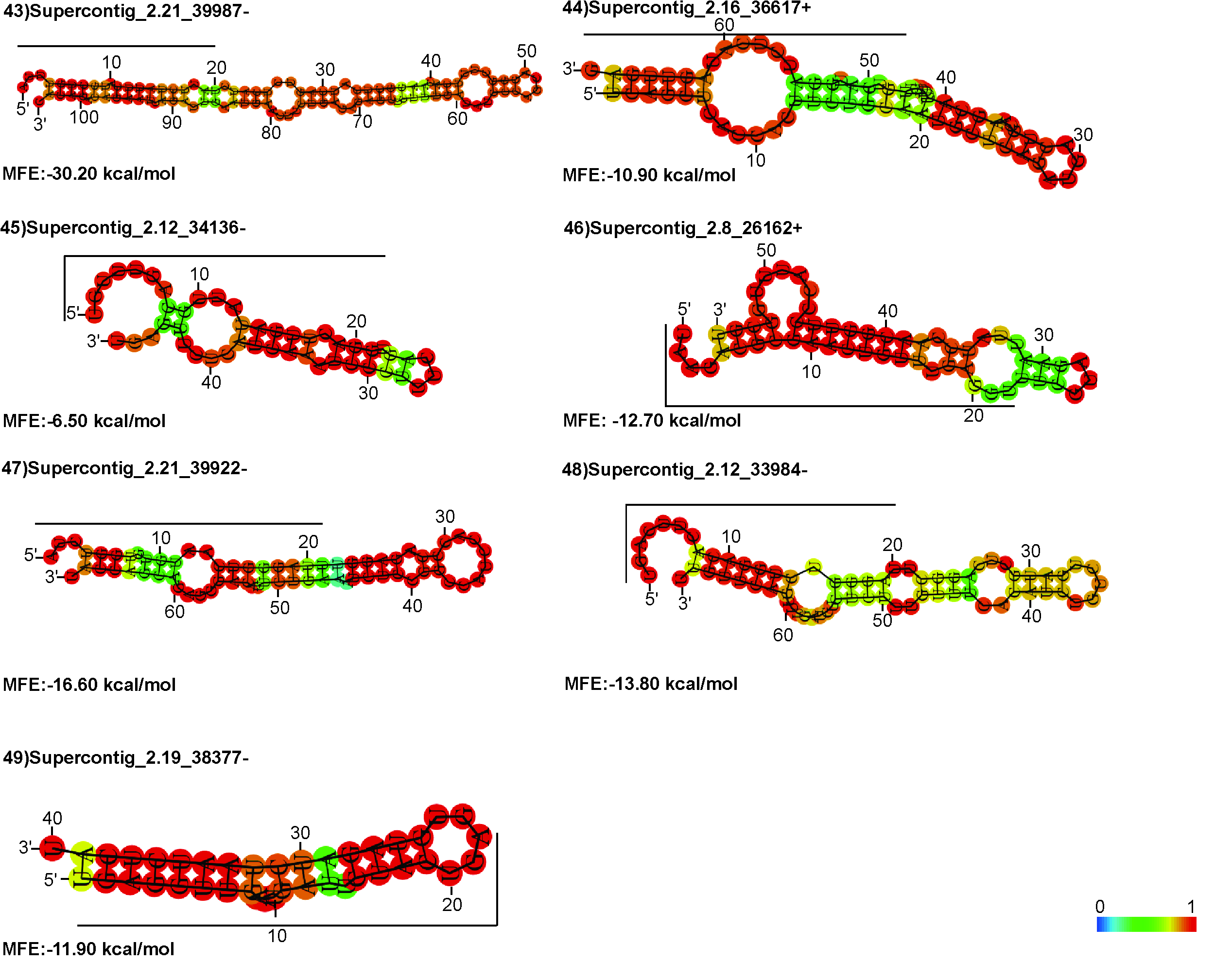

Supplement: Supplementary file 11 [file Image_7.TIF]

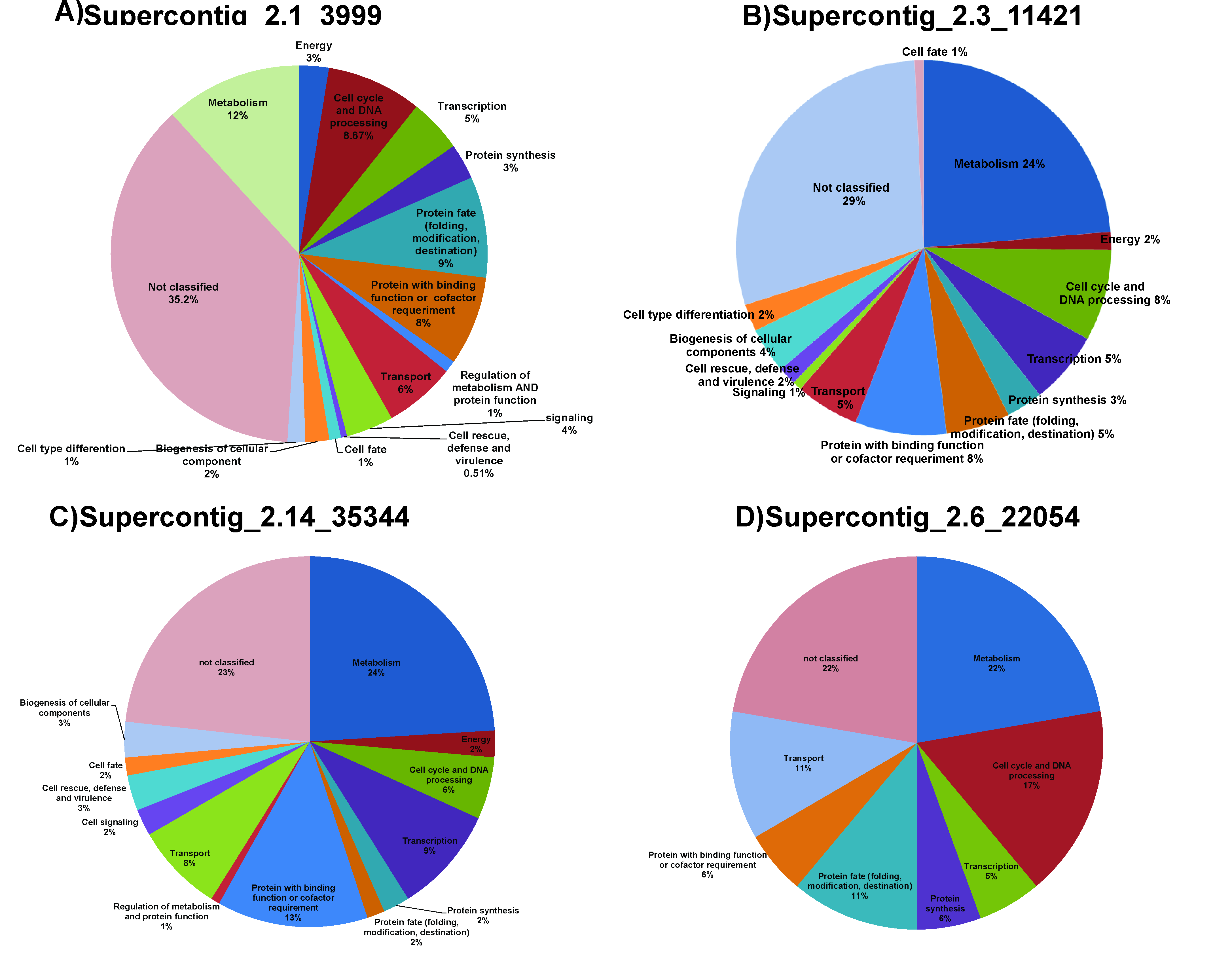

Supplement: Supplementary file 12 [file Image_8.TIF]

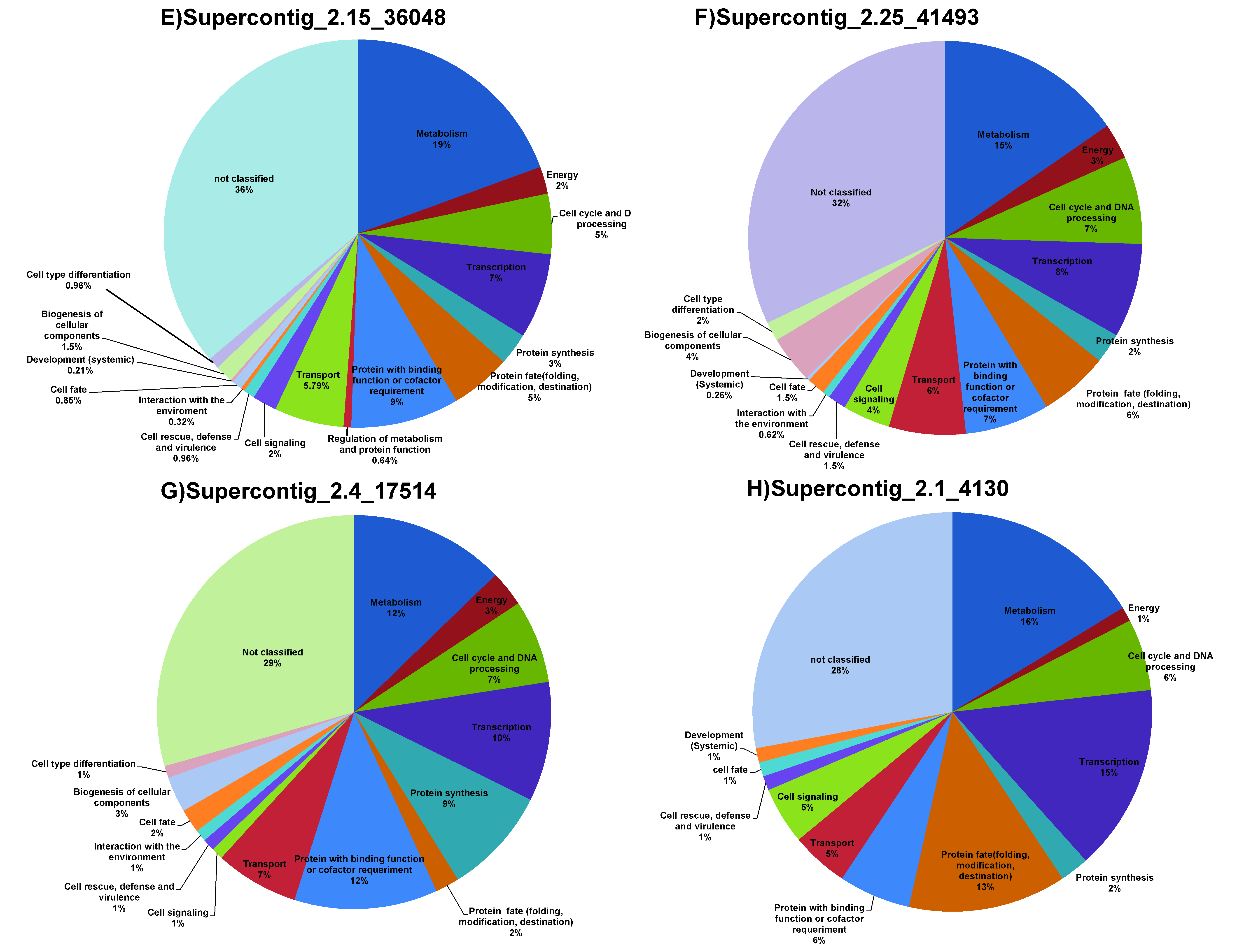

Supplement: Supplementary file 13 [file Image_9.TIF]

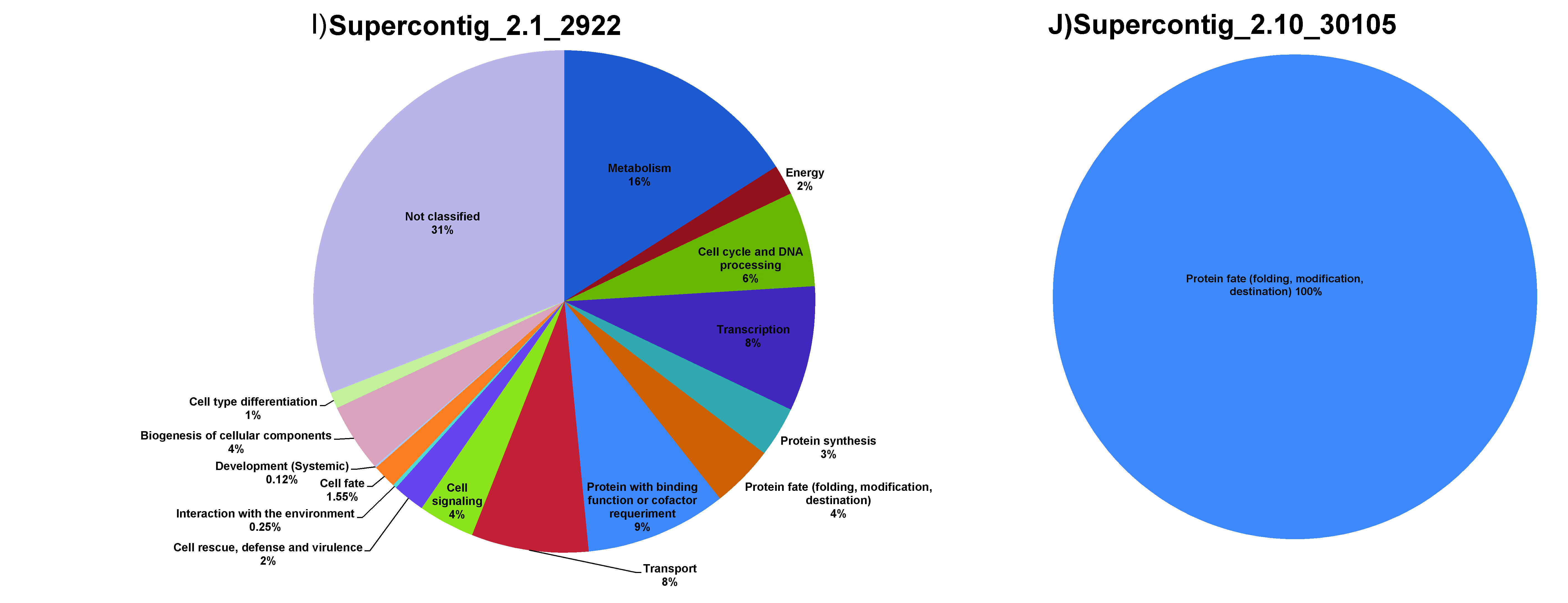

Supplement: Supplementary file 14 [file Image_10.TIF]
