# Supplementary material for: Cell Wall Synthesis, Development of Hyphae and Metabolic Pathways Are Processes Potentially Regulated by MicroRNAs Produced Between the Morphological Stages of Paracoccidioides brasiliensis
Source: Front Microbiol. 2018 Dec 11;9:3057. doi: 10.3389/fmicb.2018.03057 (PMC6297277; doi:10.3389/fmicb.2018.03057)
Supplement: Supplementary Table 1 — Oligonucleotide sequences used in the present study. [file Table_1.docx]

**Supplementary table 1- Oligonucleotide sequences used in the present study.**

| Genes | Forward sequences | Reverse sequence | | |
| --- | --- | --- | --- | --- |
| *dcr 1* | GGAGATTGAAGCTACTGAGAC | | TCTGGCAGACACTATTTACAAC |  |
| *dcr 2* | GAGGGAGGCAACCAACTATC | | TTAGAAACCACCTCGTCCTTG |  |
| *ago-1* | CCATGGCTGCAGTGTCAGTA | | AAACACCATCGCGGAAGTAGT |  |
| *ago-2* | CGACTATTTCAGACGCACATAT | | GGGTTAAGCTTAGCATTTGCC |  |
| *Act* | CGTCCTCGCCATCATGGTAT | | TCTCCATATCATCCCAGTTCG |  |
| *Endochitinase 42 kDA* | CATCGTTGAGTCTAGGCTATG | | GAGACATCCGTAAGTACCATC |  |
| *Cell wall glucanase (Scw4)* | CCATTCAGTACCCAAACCATAA | | CATCTTTCACATCAGCAGCAG |  |
| *Chitinase 3* | GCGACAGAGACGATAGATGC | | TGATGACTTGAGTGCCGGAG |  |
| *Hydrophobin 1* | AGTCTGCTCTGCTACTCTCC | | TGTGAAGAAGTAATCGATATCGT |  |
