# Supplementary material for: Cell Wall Synthesis, Development of Hyphae and Metabolic Pathways Are Processes Potentially Regulated by MicroRNAs Produced Between the Morphological Stages of Paracoccidioides brasiliensis
Source: Front Microbiol. 2018 Dec 11;9:3057. doi: 10.3389/fmicb.2018.03057 (PMC6297277; doi:10.3389/fmicb.2018.03057)
Supplement: Supplementary Table 2 — Differentially expressed microRNAs between the mycelial and transition libraries. [file Table_2.docx]

Supplementary table 2- Differentially expressed microRNAs between the mycelial and transition libraries.

| MicroRNA | log^2^FoldChange | P-value | Padj | Mycelium | Transition |
| --- | --- | --- | --- | --- | --- |
| Supercontig_2.24_41376 | 3.68526619 | 5.39E-08 | 1.32E-06 | up-regulated | down-regulated |
| Supercontig_2.13_34964 | 2.742853216 | 4.42E-05 | 0.000360582 | up-regulated | down-regulated |
| Supercontig_2.24_41413 | 1.877923863 | 1.69E-05 | 0.000275626 | up-regulated | down-regulated |
| Supercontig_2.20_39100 | 1.52208736 | 0.000395761 | 0.002154701 | up-regulated | down-regulated |
| Supercontig_2.12_33897 | 1.467721833 | 8.99E-09 | 4.41E-07 | up-regulated | down-regulated |
| Supercontig_2.27_42386 | 0.758278151 | 0.004103583 | 0.013405039 | up-regulated | down-regulated |
| Supercontig_2.12_33986 | 0.724459365 | 0.002162186 | 0.008264669 | up-regulated | down-regulated |
| Supercontig_2.34_43490 | -0.884522917 | 0.015466832 | 0.044580869 | down-regulated | up-regulated |
| Supercontig_2.21_39922 | -1.052103515 | 0.002192667 | 0.008264669 | down-regulated | up-regulated |
| Supercontig_2.12_33015 | -1.071172378 | 0.000376871 | 0.002154701 | down-regulated | up-regulated |
| Supercontig_2.1_1681 | -1.289143579 | 0.000694309 | 0.003402113 | down-regulated | up-regulated |
| Supercontig_2.1_4130 | -1.297792105 | 0.001737498 | 0.007739762 | down-regulated | up-regulated |
| Supercontig_2.10_31197 | -1.397530425 | 0.000323701 | 0.002154701 | down-regulated | up-regulated |
| Supercontig_2.4_16040 | -1.415804598 | 3.56E-05 | 0.000348634 | down-regulated | up-regulated |
| Supercontig_2.3_11421 | -1.484382105 | 0.00324939 | 0.011372864 | down-regulated | up-regulated |
| Supercontig_2.10_31175 | -1.52396594 | 3.52E-05 | 0.000348634 | down-regulated | up-regulated |
