# Supplementary material for: Cell Wall Synthesis, Development of Hyphae and Metabolic Pathways Are Processes Potentially Regulated by MicroRNAs Produced Between the Morphological Stages of Paracoccidioides brasiliensis
Source: Front Microbiol. 2018 Dec 11;9:3057. doi: 10.3389/fmicb.2018.03057 (PMC6297277; doi:10.3389/fmicb.2018.03057)
Supplement: Supplementary Table 3 — Differentially expressed microRNAs between the transition and yeast libraries. [file Table_3.docx]

Supplementary table 3- Differentially expressed microRNAs between the transition and yeast libraries.

| MicroRNAs | log^2^FoldChange | P-value | Padj | Transition | Yeast |
| --- | --- | --- | --- | --- | --- |
| Supercontig_2.3_11421 | 4.842991972 | 1.71E-05 | 3.50E-05 | up-regulated | down-regulated |
| Supercontig_2.1_3999 | 4.586334264 | 0.002885831 | 0.004159 | up-regulated | down-regulated |
| Supercontig_2.9_29703 | 3.56892224 | 8.78E-20 | 7.17E-19 | up-regulated | down-regulated |
| Supercontig_2.12_33015 | 3.559989034 | 1.60E-20 | 1.57E-19 | up-regulated | down-regulated |
| Supercontig_2.12_33986 | 2.871404261 | 1.21E-13 | 5.37E-13 | up-regulated | down-regulated |
| Supercontig_2.38_43800 | 2.673060952 | 5.05E-12 | 1.77E-11 | up-regulated | down-regulated |
| Supercontig_2.21_39922 | 2.633295669 | 5.17E-13 | 2.11E-12 | up-regulated | down-regulated |
| Supercontig_2.21_39987 | 2.491237442 | 4.46E-14 | 2.43E-13 | up-regulated | down-regulated |
| Supercontig_2.19_38665 | 2.151157228 | 8.65E-11 | 2.82E-10 | up-regulated | down-regulated |
| Supercontig_2.20_39013 | 1.884230016 | 1.65E-06 | 3.85E-06 | up-regulated | down-regulated |
| Supercontig_2.12_33984 | 1.814350811 | 6.85E-06 | 1.46E-05 | up-regulated | down-regulated |
| Supercontig_2.27_42386 | 1.378717587 | 4.94E-05 | 8.65E-05 | up-regulated | down-regulated |
| Supercontig_2.22_40198 | 1.342383031 | 0.000358179 | 0.000532 | up-regulated | down-regulated |
| Supercontig_2.5_19199 | 1.281489704 | 2.50E-05 | 4.71E-05 | up-regulated | down-regulated |
| Supercontig_2.5_19191 | 1.088019598 | 0.000326243 | 0.0005 | up-regulated | down-regulated |
| Supercontig_2.19_38040 | 1.048697659 | 0.000279322 | 0.000442 | up-regulated | down-regulated |
| Supercontig_2.19_38377 | 1.048697659 | 0.000279322 | 0.000442 | up-regulated | down-regulated |
| Supercontig_2.12_33897 | 0.767891165 | 0.019761828 | 0.025482 | up-regulated | down-regulated |
| Supercontig_2.5_19148 | -1.379826126 | 4.50E-05 | 8.16E-05 | down-regulated | up-regulated |
| Supercontig_2.10_31175 | -1.799305845 | 1.49E-06 | 3.66E-06 | down-regulated | up-regulated |
| Supercontig_2.24_41413 | -2.258741535 | 3.54E-07 | 9.64E-07 | down-regulated | up-regulated |
| Supercontig_2.2_5957 | -2.506802813 | 1.29E-12 | 4.85E-12 | down-regulated | up-regulated |
| Supercontig_2.9_29173 | -2.624338846 | 0.005765853 | 0.007848 | down-regulated | up-regulated |
| Supercontig_2.28_42699 | -2.65450368 | 6.80E-06 | 1.46E-05 | down-regulated | up-regulated |
| Supercontig_2.10_30363 | -2.768651883 | 0.003964359 | 0.00555 | down-regulated | up-regulated |
| Supercontig_2.45_44026 | -2.963340515 | 0.037644752 | 0.047297 | down-regulated | up-regulated |
| Supercontig_2.1_4130 | -3.341420956 | 9.37E-16 | 6.56E-15 | down-regulated | up-regulated |
| Supercontig_2.9_28895 | -3.413511419 | 1.81E-41 | 2.96E-40 | down-regulated | up-regulated |
| Supercontig_2.25_41493 | -3.954881202 | 6.78E-40 | 8.31E-39 | down-regulated | up-regulated |
| Supercontig_2.24_41376 | -4.360254307 | 3.22E-07 | 9.27E-07 | down-regulated | up-regulated |
| Supercontig_2.8_26162 | -4.412715167 | 1.95E-05 | 3.82E-05 | down-regulated | up-regulated |
| Supercontig_2.6_22054 | -4.588081284 | 1.52E-09 | 4.65E-09 | down-regulated | up-regulated |
| Supercontig_2.27_42084 | -5.653748698 | 8.79E-05 | 0.000148 | down-regulated | up-regulated |
| Supercontig_2.15_36048 | -6.025314084 | 8.27E-48 | 2.03E-46 | down-regulated | up-regulated |
| Supercontig_2.1_2922 | -6.840924204 | 8.47E-14 | 4.15E-13 | down-regulated | up-regulated |
| Supercontig_2.10_30105 | -8.005471413 | 1.12E-14 | 6.84E-14 | down-regulated | up-regulated |
| Supercontig_2.4_17514 | -8.053057542 | 3.08E-61 | 1.51E-59 | down-regulated | up-regulated |
| Supercontig_2.14_35344 | -8.405374165 | 4.35E-07 | 1.12E-06 | down-regulated | up-regulated |
