# Supplementary material for: Cell Wall Synthesis, Development of Hyphae and Metabolic Pathways Are Processes Potentially Regulated by MicroRNAs Produced Between the Morphological Stages of Paracoccidioides brasiliensis
Source: Front Microbiol. 2018 Dec 11;9:3057. doi: 10.3389/fmicb.2018.03057 (PMC6297277; doi:10.3389/fmicb.2018.03057)
Supplement: Supplementary Table 4 — Differentially expressed microRNAs between the mycelium and yeast libraries. [file Table_4.DOCX]

Supplementary table 4- Differentially expressed microRNAs between the mycelium and yeast libraries.

| MicroRNAs | log^2^Fold Change | P-value | Padj | Mycelium | Yeast |
| --- | --- | --- | --- | --- | --- |
| Supercontig_2.1_3999 | 7.105539883 | 3.90E-06 | 9.09E-06 | up-regulated | down-regulated |
| Supercontig_2.13_34964 | 3.766658482 | 0.001721 | 0.002635 | up-regulated | down-regulated |
| Supercontig_2.12_33986 | 3.595863626 | 1.55E-20 | 1.26E-19 | up-regulated | down-regulated |
| Supercontig_2.3_11421 | 3.358609866 | 0.002898 | 0.004303 | up-regulated | down-regulated |
| Supercontig_2.9_29703 | 3.328281386 | 2.09E-17 | 1.28E-16 | up-regulated | down-regulated |
| Supercontig_2.38_43800 | 2.873597128 | 1.15E-13 | 5.14E-13 | up-regulated | down-regulated |
| Supercontig_2.12_33015 | 2.488816656 | 8.61E-11 | 2.81E-10 | up-regulated | down-regulated |
| Supercontig_2.21_39987 | 2.292888588 | 3.79E-12 | 1.55E-11 | up-regulated | down-regulated |
| Supercontig_2.12_33897 | 2.235612998 | 1.09E-11 | 3.82E-11 | up-regulated | down-regulated |
| Supercontig_2.20_39013 | 2.165306965 | 3.65E-08 | 9.93E-08 | up-regulated | down-regulated |
| Supercontig_2.27_42386 | 2.136995738 | 3.12E-10 | 9.54E-10 | up-regulated | down-regulated |
| Supercontig_2.19_38665 | 1.748336489 | 1.35E-07 | 3.48E-07 | up-regulated | down-regulated |
| Supercontig_2.21_39922 | 1.581192154 | 1.46E-05 | 3.25E-05 | up-regulated | down-regulated |
| Supercontig_2.22_40198 | 1.515221863 | 5.61E-05 | 0.000119 | up-regulated | down-regulated |
| Supercontig_2.20_39100 | 1.27630446 | 0.022857 | 0.028717 | up-regulated | down-regulated |
| Supercontig_2.5_19191 | 1.182651353 | 9.40E-05 | 0.000177 | up-regulated | down-regulated |
| Supercontig_2.12_33984 | 0.998962297 | 0.013267 | 0.01757 | up-regulated | down-regulated |
| Supercontig_2.19_38040 | 0.947519019 | 0.001028 | 0.00168 | up-regulated | down-regulated |
| Supercontig_2.19_38377 | 0.947519019 | 0.001028 | 0.00168 | up-regulated | down-regulated |
| Supercontig_2.5_19199 | 0.85284238 | 0.00506 | 0.007084 | up-regulated | down-regulated |
| Supercontig_2.4_16040 | -1.034768702 | 0.018781 | 0.024218 | down-regulated | up-regulated |
| Supercontig_2.5_19148 | -1.357115575 | 6.05E-05 | 0.000119 | down-regulated | up-regulated |
| Supercontig_2.1_1681 | -1.746689046 | 5.94E-05 | 0.000119 | down-regulated | up-regulated |
| Supercontig_2.10_31197 | -2.085442673 | 1.46E-07 | 3.57E-07 | down-regulated | up-regulated |
| Supercontig_2.28_42699 | -2.155123246 | 0.000248 | 0.00045 | down-regulated | up-regulated |
| Supercontig_2.2_5957 | -2.403349581 | 1.03E-11 | 3.82E-11 | down-regulated | up-regulated |
| Supercontig_2.10_30363 | -2.764054592 | 0.00417 | 0.006009 | down-regulated | up-regulated |
| Supercontig_2.8_26162 | -3.322413362 | 0.001303 | 0.002059 | down-regulated | up-regulated |
| Supercontig_2.10_31175 | -3.323271785 | 6.99E-19 | 4.89E-18 | down-regulated | up-regulated |
| Supercontig_2.9_28895 | -3.519352138 | 9.74E-44 | 1.59E-42 | down-regulated | up-regulated |
| Supercontig_2.27_42084 | -3.876847871 | 0.006697 | 0.009115 | down-regulated | up-regulated |
| Supercontig_2.25_41493 | -4.130461472 | 6.59E-43 | 8.07E-42 | down-regulated | up-regulated |
| Supercontig_2.1_4130 | -4.639213062 | 7.57E-29 | 7.42E-28 | down-regulated | up-regulated |
| Supercontig_2.6_22054 | -4.685124996 | 9.82E-10 | 2.83E-09 | down-regulated | up-regulated |
| Supercontig_2.14_35344 | -5.592115216 | 0.000433 | 0.000759 | down-regulated | up-regulated |
| Supercontig_2.15_36048 | -6.203401859 | 2.31E-50 | 5.67E-49 | down-regulated | up-regulated |
| Supercontig_2.1_2922 | -7.651258173 | 2.32E-16 | 1.26E-15 | down-regulated | up-regulated |
| Supercontig_2.4_17514 | -8.946602967 | 3.34E-71 | 1.64E-69 | down-regulated | up-regulated |
| Supercontig_2.10_30105 | -9.782396983 | 1.09E-14 | 5.34E-14 | down-regulated | up-regulated |
